# Alterations of Cell Proliferation and Apoptosis in the Hypoplastic *Reeler* Cerebellum

**DOI:** 10.3389/fncel.2016.00141

**Published:** 2016-05-25

**Authors:** Carolina Cocito, Adalberto Merighi, Mario Giacobini, Laura Lossi

**Affiliations:** ^1^Laboratory of Neuroscience, Department of Veterinary Sciences, University of TurinGrugliasco, Italy; ^2^Laboratory of Dynamical Systems and Epidemiology, Department of Veterinary Sciences, University of TurinGrugliasco, Italy

**Keywords:** *Reeler*, cerebellum, mouse, cell proliferation, apoptosis, programmed cell death, development, neurochemistry

## Abstract

A mutation of the *reln* gene gives rise to the *Reeler* mouse (*reln*^−∕−^) displaying an ataxic phenotype and cerebellar hypoplasia. We have characterized the neurochemistry of postnatal (P0–P60) *reln*^−∕−^ mouse cerebella with specific attention to the intervention of cell proliferation and apoptosis in the P0–P25 interval. Homozygous *reln*^−∕−^ mice and age-matched controls were analyzed by immunofluorescence using primary antibodies against NeuN, calbindin, GFAP, vimentin, SMI32, and GAD67. Proliferation and apoptosis were detected after a single intraperitoneal BrdU injection and by the TUNEL assay with anti-digoxigenin rhodamine-conjugated antibodies. Quantitative analysis with descriptive and predictive statistics was used to calculate cell densities (number/mm^2^) after fluorescent nuclear stain (TCD, total cell density), labeling with BrdU (PrCD, proliferating cell density), or TUNEL (ApoCD, apoptotic cell density). By this approach we first have shown that the temporal pattern of expression of neuronal/glial markers in postnatal cerebellum is not affected by the *Reeler* mutation. Then, we have demonstrated that the hypoplasia in the *Reeler* mouse cerebellum is consequent to reduction of cortical size and cellularity (TCD), and that TCD is, in turn, linked to quantitative differences in the extent of cell proliferation and apoptosis, as well as derangements in their temporal trends during postnatal maturation. Finally, we have calculated that PrCD is the most important predictive factor to determine TCD in the cerebellar cortex of the mutants. These results support the notion that, beside the well-known consequences onto the migration of the cerebellar neurons, the lack of Reelin results in a measurable deficit in neural proliferation.

## Introduction

The cerebellum is one of the main parts of the brain involved in motor coordination. It consists of a highly ordered layered cortex made of gray matter at its surface, and a deep central medullary body made of white matter that spreads radially to form a typical arborization in reaching the axis of individual cerebellar laminae (folia). Embedded into the medullary body are three (four in humans) nuclei of neurons giving rise to the majority of the cerebellar efferent fibers. In mice and other altricial mammals—such as humans—most of cerebellar development occurs postnatally (Altman and Bayer, 1997). Postnatal development of the cerebellar cortex consists of a very complex series of tightly regulated events leading to the generation of the granule cells and the GABAergic cortical interneurons. These cells, as well as the other cortical neurons and the glia, have to perform very long distance migration from their progenitor niches, and neurons extend their axons for making highly precise synaptic connections with targets (Marzban et al., 2015). Until the second week after birth in rodents, extensive neurogenesis within a temporary external granular layer (EGL) gives rise to the granule cells that migrate through an increasingly thick molecular layer (ML) and the forming Purkinje cell layer. The granule cells finally reach the internal granular layer (IGL), which, eventually, will be the only granular layer of the mature cerebellar cortex (Altman, 1972a,b; Hatten, 1990). Notably, postnatal neurogenesis in cerebellum accompanies with an intense programmed cell death to attain a numerical match between cortical neurons, mainly the granule cells, and the Purkinje neurons. Several types of death may affect neurons, and among them apoptosis (Kerr et al., 1972), whose key features are DNA fragmentation, chromatin condensation, cell shrinkage and/or fragmentation, and activation of specific cellular proteases (Yamaguchi and Miura, 2015). The granule cells undergo apoptosis in two different phases of their differentiation, maturation, and migration: one phase occurs in the EGL and is independent from synapse formation, the latter takes place in the IGL, and depends on the classical mechanisms of neuron-to-target interaction at synapses. The first phase of granule cell apoptosis is rapid, and necessary for a gross regulation of the expanding pre-mitotic population of these neurons; the second occurs in a longer interval of time, and corresponds to the period of synaptic sculpting of the cerebellar cortex (Lossi et al., 2002). Wiring of the connections between the granule cells and the Purkinje neurons is fundamental to cerebellar function (Hirai and Launey, 2000; Cesa and Strata, 2009), and it is unanimously agreed that these two neuronal populations exert reciprocal influences that are fundamental in the regulation of their survival or death (Altman, 1992). Therefore, the maintenance of a correct balance between neurogenesis and apoptosis is essential for proper cerebellar maturation, to the point that such a balance is deeply altered in several spontaneous mutations affecting the murine cerebellum and leading to ataxias (Cendelin, 2014).

*Reeler* is the first described mouse cerebellar mutation (Falconer, 1951). The *Reeler* phenotype is characterized by typical alterations in gait (“reeling”) and was named thereafter. The recessive homozygous mouse (*reln*^−∕−^) displays a total absence of Reelin, a protein discovered several decades after the initial description of the mutant (D'Arcangelo et al., 1995). Reelin is necessary for brain development as it plays a prominent role in neuronal migration. During cerebellar development, granule cells in the EGL secrete the protein, which is fundamental for the proper migration and positioning of the Purkinje neurons (D'Arcangelo et al., 1995). The role of Reelin in the adulthood is still under debate, but many authors demonstrated, among others, that it intervenes in the growth of apical dendrites, the formation of dendritic spines, and the regulation of synaptic function and plasticity (D'Arcangelo, 2014). In *reln*^−∕−^ mice, the lack of Reelin leads to a severe hypoplasia of cerebellum. Histologically, *reln*^−∕−^mice display a thinner ML, a reduction in the density of the granule cells in the IGL, and a misplacement of the Purkinje neurons. Just 5% of these neurons align into their normal position, 10% are located in the IGL, and the remaining ones are scattered in a central deeper mass inside the white matter of the medullary body (Mariani et al., 1977; Heckroth et al., 1989; Yuasa et al., 1993). Despite these obvious alterations, part of the cerebellar circuitry is maintained, but, as demonstrated by several studies, at the expenses of a drastic reduction in the number of synapses between the Purkinje neurons and the parallel fibers, and of an altered pruning of the climbing fibers-Purkinje neurons synapses (Mariani et al., 1977; Qiao et al., 2013; Castagna et al., 2014). In keeping with these observations, other studies highlighted how the lack of Reelin also results in impaired hippocampal postnatal neurogenesis and functionality (Won et al., 2006; Pujadas et al., 2010).

Work on the *Reeler* mutation has focused onto the intracellular pathways of signal transduction mediated by Reelin and the cellular and molecular mechanisms that are affected by its deficiency (see D'Arcangelo, 2014 for a recent review). Surprisingly, little or no attention was devoted to the relationship between neurogenesis and programmed cell death in the course of postnatal cerebellar development. In this study, we have investigated such a relationship and quantitatively demonstrated an impairment of postnatal neurogenesis in *reln*^−∕−^ mice, in parallel with an increase in apoptosis. In addition, as previous ultrastructural observations from our group showed a high degree of immaturity in *reln*^−∕−^ Purkinje neurons' synaptic circuitry (Castagna et al., 2014) we have compared the timing of expression of several markers of neurochemical differentiation across the mutants and the normal mice. This had the purpose to exclude that substantial temporal differences in the acquisition of specific phenotypic features of the different cerebellar neural cells biased our analysis. As no obvious neurochemical differences emerged between the two genotypes, our data show, for the first time, the occurrence of precise imbalances in the ratio of neurogenesis to apoptosis in the *Reeler* mouse, and further prove the contribution of Reelin in neuronal survival.

## Materials and methods

### Animals

Mouse cerebella at postnatal (P) day 0, 5, 10, 15, 20, 25, 30, and 60 from *reln*^+∕+^ and *reln*^−∕−^ mice were compared in this study (n = three each). The number of animals was kept to a minimum, and all efforts were made to minimize their suffering. The Italian Ministry of Health (#65.2016.PR) and the Bioethics Committee of the University of Turin authorized all experiments. Animal procedures were carried out according to the guidelines and recommendations of the European Union (Directive 2010/63/UE) as implemented by current Italian regulations on animal welfare (DL n. 26-04/03/2014).

### Genotyping

All animals were genotyped according to current protocols to ascertain their genetic background. Briefly, a small sample was cut from the tail tip and incubated overnight with continuous shaking in a solution containing 50 μg/mL proteinase K (Sigma) in lysis buffer (Tween 20, NonIDET P40, 1% gelatin, KCl 1M, Tris 1M pH 8.5, MgCl2 0.5M). On the following morning, samples were centrifuged at 14,000 g for 5 min and the DNA-containing surnatant used for PCR amplification. The gene mutation (*reln*^−∕−^) in the *Reeler* phenotype is responsible for deletion of part of an intron having control over correct gene expression and stability of the mRNA (D'Arcangelo et al., 1996). To identify the three possible genotypes in littermates (*reln*^+∕+^, *reln*^+∕−^, and *reln*^−∕−^), a sequence containing the potentially deleted one is amplified by PCR using three different primers. Primers were a common forward primer within the conserved protein sequence upstream the mutation, and two different reverse primers, one of which is specific for the wild type sequence and the other for a sequence downstream the mutation. By this procedure, the band obtained from amplification of the cDNA for the wild type Reelin has a molecular weight of 266 bp, and that obtained for the mutated Reelin weighs 363 bp. After electrophoresis and ethidium bromide staining in 1.5% agarose gel, mice were classified as follows: i. Wild type controls (*reln*^+∕+^) when displaying a single 266 bp band, ii. *Reeler* (*reln*^−∕−^) when displaying a single 363 bp band, and iii. Heterozygous (*reln*^+∕−^) when displaying both bands.

### Histology

All procedures were carried out at room temperature, unless otherwise stated. Two hours before the sacrifice, mice were injected with 5-bromo-2′-deoxyuridine (BrdU) intraperitoneally (0.1 mg/g body weight). Under deep anesthesia (sodium pentobarbital 30 mg/kg), animals then were perfused through the left ventricle with cold Ringer solution (0.01 M phosphate buffer pH 7.4–7.6, 0.8% NaCl, 0.025% KCl, 0.05% NaHCO_3_) followed by fixative (4% paraformaldehyde in phosphate buffer 0.2 M pH 7.4). Cerebella were removed and post-fixed for 2 h in the same fixative. Tissues were then dehydrated through a graded ethanol series and embedded in paraffin wax. The entire cerebellum was serially cut in parasagittal sections (7 μm) that were collected and mounted on poly-L-lysine (PLL; Sigma Aldrich) pre-coated slides (5 sections/slide).

For immunohistochemistry, sections were rehydrated and subjected to microwave antigen retrieval (9 min at 95–99°C in sodium citrate buffer pH 6). After microwave treatment, sections where washed 5 min in phosphate buffered saline 0.01 M pH 7.4 (PBS) and blocked for 1 h in PBS containing 1% ovoalbumin (Sigma Aldrich) and 0.3% Triton X-100 (Sigma Aldrich). Sections were incubated overnight in primary antibodies at optimal titer, with the exception of BrdU immunostaining where incubation was carried out for 1 h only. After being rinsed 3 × 5 min in PBS, sections were incubated for 1 h with fluorescent secondary antibody conjugates (anti-rabbit or anti-mouse Alexa Fluor® 488 or Alexa Fluor® 594, Molecular Probes, Life Technology) diluted 1:800 in the same diluent used for primary antibodies. When necessary, nuclear counterstaining with propidium iodide (PI, Sigma Aldrich) was applied (1 μg/mL in PBS containing 0.3% Triton X100, 2 min), and slices were eventually mounted in antifade medium (Sigma Aldrich).

#### Primary antibodies

Primary antibodies were: mouse anti-NeuN (Millipore, 1:200); rabbit anti-vimentin (Cell Signaling, 1:100); mouse anti-calbindin (ABCAM, 1:300); rabbit anti-GFAP (ABCAM, 1:2000); mouse anti-Smi32 (ABCAM, 1:1500); rabbit anti-GAD67 (Ana Spec, 1:100); mouse anti-BrdU (GE Healthcare, prediluted in nuclease solution). Primary antibodies were diluted in PBS containing 2% bovine serum albumin (BSA; Sigma Aldrich) and 2% PLL. Routine specificity and method controls were performed by omission of primary antibodies, their substitution with normal serum, omission of secondary antibodies or their substitution with inappropriate species-specific conjugates.

#### Analysis of apoptosis

*In situ* labeling of cells with fragmented DNA was performed following a modification of the original terminal dUTP nick end labeling (TUNEL) procedure (Gavrieli et al., 1992). In brief, after being brought to double distilled water (ddH_2_O), sections were pretreated with proteinase K (Sigma Aldrich) for 15 min at 37°C in a humid atmosphere. The proteinase K working solution (20 μg/mL) was prepared from a stock solution of proteinase K (1 mg/mL in 10 mM Tris pH 7.7) by mixing together 80% Tris 10 mM pH 7.7, 18% 1 mM CaCl_2_, and 2% proteinase K stock solution. After 4 × 2 min washings in ddH_2_O, sections were immersed for 10 min in TdT buffer (30 mM Tris-HCl, pH 7, 140 mM sodium cacodylate, 1 mM cobalt chloride) and subsequently incubated in TdT buffer supplemented with 0.05 U/μL terminal transferase and 10 μM digoxigenin-dUTP at 37°C for two and half hours in a humid atmosphere. The reaction was stopped by transferring the sections to terminal buffer (300 mM sodium chloride, 30 mM sodium citrate in ddH_2_O) for 15 min. Sections were subsequently rinsed in ddH_2_O, blocked in 2.5% BSA in ddH_2_O for 10 min, and incubated overnight at 4°C with a rabbit anti-digoxigenin antibody conjugated to rhodamine (Molecular Probes, Life Technology) diluted 1:75 in PBS/PLL/BSA. After extensive washing in PBS, sections were counterstained with 1:1000 4′, 6-diamidino-2-phenylindole (DAPI—Sigma Aldrich) in PBS for 2 min and finally mounted in antifade medium.

### Quantitative analysis of proliferating and apoptotic cells

#### Sampling strategy

The whole series of sections cut through the entire latero-lateral (transversal) axis of cerebellum was divided into ten sampling units. The total number of sections for each sampling unit then was related to the overall size of the cerebellum. Following a systematic random sampling procedure (Geuna, 2000), two slides were arbitrarily selected within each sampling unit, one to be processed for BrdU immunostaining (*n* = 5 sections) and the other according to the TUNEL protocol (*n* = 5 sections). Therefore, fifty randomly selected sections were processed to gather quantitative data on BrdU or TUNEL labeled cells. Five additional sections were selected at random, stained with PI and used for cell density studies as described in Section Calculation of Total Cell Density (TCD) in Single Layers of Cerebellar Cortex and Medullary Body.

#### Image acquisition

A 20x lens and a transmitted/fluorescence light microscope equipped with appropriate filter combinations to detect rhodamine and DAPI (DM6000B, Leica) were used to collect wide-field fluorescence microscopy images (1392 × 1040 pixels). Laser scanning confocal images (1024 × 1024 pixels) were collected with 40x dry lens and a laser scanning confocal microscope (LSCM—SP5, Leica) using the laser excitation lines required by Alexa Fluor® 488, Alexa Fluor® 594, or PI.

#### Identification of cortical layers and medullary body

**Figure 1:**
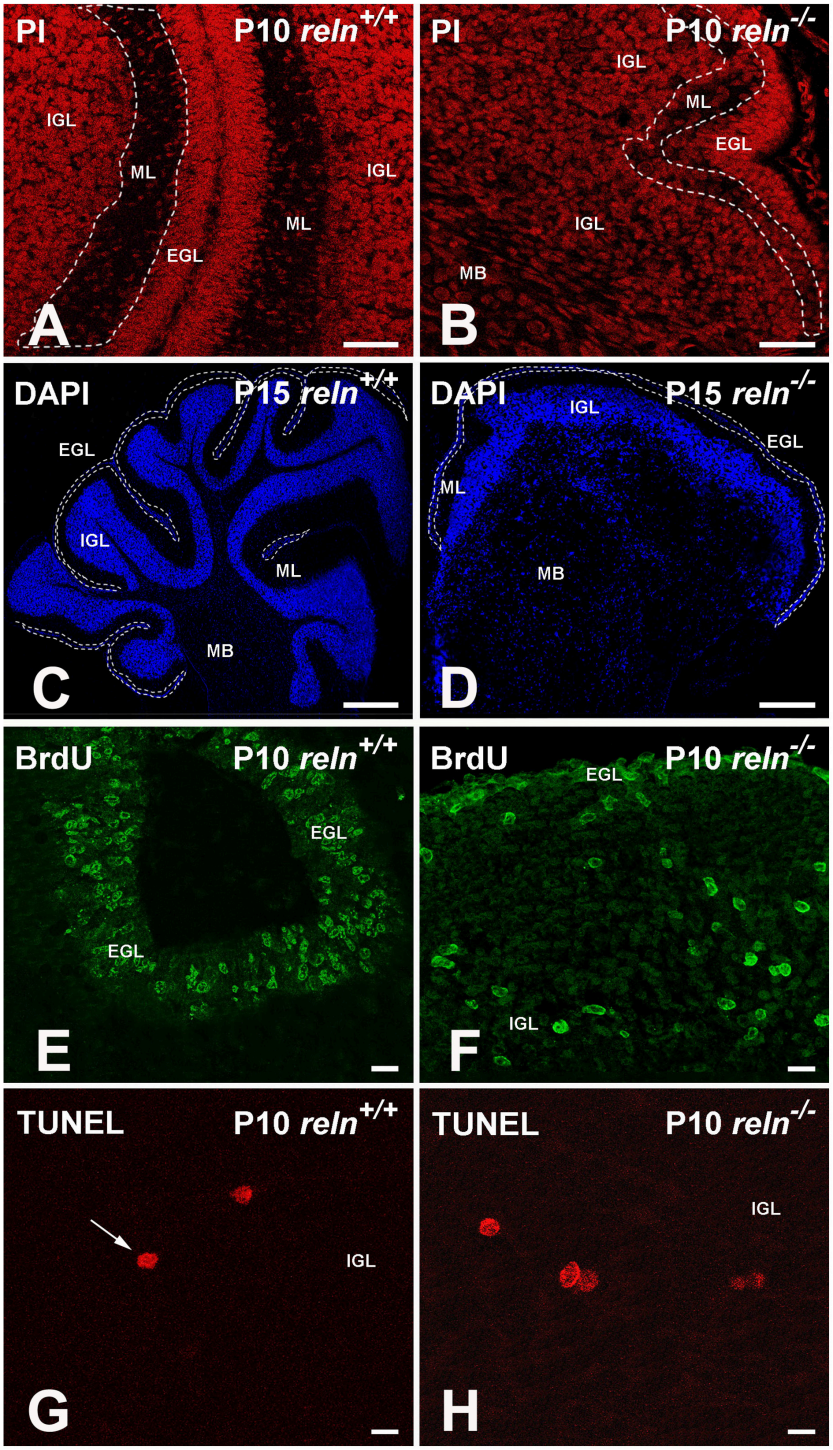
**Exemplificative images to explain the histological procedures described in Material and methods. (A,B)** Propidium iodide (PI) staining was used to calculate TCD in EGL, ML, IGL and medullary body. The ML is delimited by the white dashed lines; **(C,D)** Reconstruction of cerebellar slices. DAPI staining allowed the identification and the measurement of the area of each cortical layer and of the medullary body. As an example of the procedure, the borders of the EGL were traced with a white dashed line. Compare the two images to easily appreciate the difference in the cytoarchitecture between the two genotypes. **(E,F)** BrdU immunostaining. Mice were sacrificed 2 h after the BrdU injection. The EGL has the higher density of proliferating cells in both genotypes, but in *reln* ^−∕−^ mice its extension is clearly reduced. **(G,H)** TUNEL^+^ nuclei in the IGL are intensely fluorescent and display different staining intensities. A condensed nucleus in G is indicated by the arrow. EGL, external granular layer; IGL, internal granular layer; MB, medullary body; ML, molecular layer; P, postnatal age. Scale bars: **(A,B)** = 25 μm; **(C,D)** = 50 μm; **(E,F)** = 10 μm; **(G,H)** = 100 μm.

The well-known alterations in neuronal migration that characterize the *Reeler* histological phenotype were taken into account to subdivide cerebellar sections into discrete areas when moving from the pial surface to the medullary body. Therefore, on cytoarchitectonic features, we could easily distinguish the EGL and the ML that displayed similar features in *reln*^+∕+^ and *reln*^−∕−^ mice. However, in the *reln*^−∕−^ genotype, impaired migration of the Purkinje neurons in the course of the physiological process of their alignment to a cell monolayer results in the lack of a true Purkinje cell layer in the mutants. Therefore, the Purkinje cell layer (only present in *reln*^+∕+^ mice) and the IGL were considered together and simply referred to as the IGL in the following. The term medullary body was used in its *bona fide* significance for *reln*^+∕+^ animals, and to indicate the inner central mass of non-migrated neurons intermingled with the white matter that characterizes the histology of the deep cerebellum in *reln*^−∕−^ mutants (Figure 1).

#### Calculation of total cell density (TCD) in single layers of cerebellar cortex and medullary body

Five randomly selected areas from each of the three cortical layers and the medullary body were sampled from PI stained sections (Figures 1A,B). Confocal images were acquired and used to calculate TCD (n. cells/mm^2^) with the Count Particle command of the Image J software (http://imagej.nih.gov/ij/) after appropriate scale and threshold setting.

#### Calculation of the density of BrdU^+^ and TUNEL^+^ cells in single layers of cerebellar cortex and medullary body

##### BrdU^+^ cell

In each cortical layer and the medullary body, we have calculated the density of BrdU^+^ cells (n. cells/mm^2^) as an index of cell proliferation, and thus herein referred to it as PrCD = proliferating cell density. To do so, we first calculated the relative percentages of the areas of each cortical layer and of the medullary body vs. the total area of the section after staining with DAPI (Figures 1C,D) and observation in the wide-field fluorescence microscope. Then, we randomly acquired five LSCM snapshot images from each of the BrdU-immunostained sections (sampled as described in Section Sampling Strategy). As exemplified in Figures 1E,F, the relatively high number of IR cells permitted an easy identification of the three cortical layers and the medullary body by simple histological landmarks and in the absence of nuclear counterstain. For each digital image, PrCD was measured in the four cerebellar subdivisions with the Image J software, as described in Section Calculation of Total Cell Density (TCD) in Single Layers of Cerebellar Cortex and Medullary Body. Finally, measures were corrected according to the ratio between each of the areas and the total area of the section, exemplified as PrCD_EGL_ = PrCD${}_{\left(BrdU\right)}^{*}$[EGL area_(*DAPI*)_/total section area_(*DAPI*)_].

##### TUNEL^+^ cell

In each cortical layer and the medullary body we have calculated the density of TUNEL^+^ cells (n. cells/mm^2^) as an index of apoptosis, and thus herein referred to it as ApoCD = apoptotic cell density. As TUNEL^+^ cells were by far less abundant than BrdU^+^ cells (Figures 1G,H), the procedure described in Section BrdU^+^ cell was prone to introduce an error too high. Therefore, to calculate ApoCD, we directly counted *all* TUNEL^+^ cells in cerebellar sections that were reconstructed from individual wide-field fluorescence images after DAPI nuclear counterstain. Reconstructions were made with the Adobe Photoshop CS6 software (Adobe Systems). At the same time, in reconstructed images, we measured the areas of each of the three cortical layers and the medullary body, and cell densities were calculated directly.

##### Normalization of BrdU^+^ and TUNEL^+^ cell densities according to genotype

Significant differences emerged from the comparison of the areas of cortical layers and medullary body between the two genotypes. Therefore, densities of each compartment in *reln*^−∕−^ mice were normalized to values in normal mice by correcting per area ratios (*reln*^−∕−^ areas/*reln*^+∕+^ areas).

#### Cell percentages

The percentages of BrdU^+^ and TUNEL^+^ cells were obtained by calculating the ratios of PrCDs or ApoCDs and TCDs for each of the cerebellar subdivisions defined in Section Identification of Cortical Layers and Medullary Body.

#### Statistics

The role of raw TCD, PrCD, and Apo CD with respect to the *reln* ^+∕+^ and *reln*^−∕−^ groups was investigated by within-subjects, i.e., repeated-measures, analysis of variance (ANOVA) with the R open-source software (R version 3.2.0, http://www.R-project.org/). Each feature was considered as an independent categorical variable, with the animal ID as within-subject factor for the observation grouping. Observed independent variables were considered significantly correlated to the outcome when the *P*-value associated to the F statistics was found to be smaller than 0.05.

With GraphPad Prism (GraphPad Software), areas and percentages of BrdU^+^ and TUNEL^+^ cells were compared with parametric Student T Test and nonparametric Mann Whitney test. Normality was ascertained with D'Agostino-Pearson omnibus normality test. Using a one-way ANOVA with Tukey correction for multiple comparisons, normalized TCD, PrCD, and ApoCD were compared at different postnatal ages within each genotype.

Mean values of TCD, PrCD, and ApoCD in spreadsheet were finally used to calculate Pearson's correlation curves and polynomial models for time effects, by plotting time (independent variable) vs. TCD, PrCD, or Apo CD (dependent variables) with the Microsoft Excel graph function. TCD, PrCD, and Apo CD mean values were also used for multiple regression analysis and statistics using the Microsoft Excel data analysis tool pack.

## Results

### The temporal pattern of expression of the main neuronal/glial markers in postnatal cerebellum is not affected by the *Reeler* mutation

**Figure 2:**
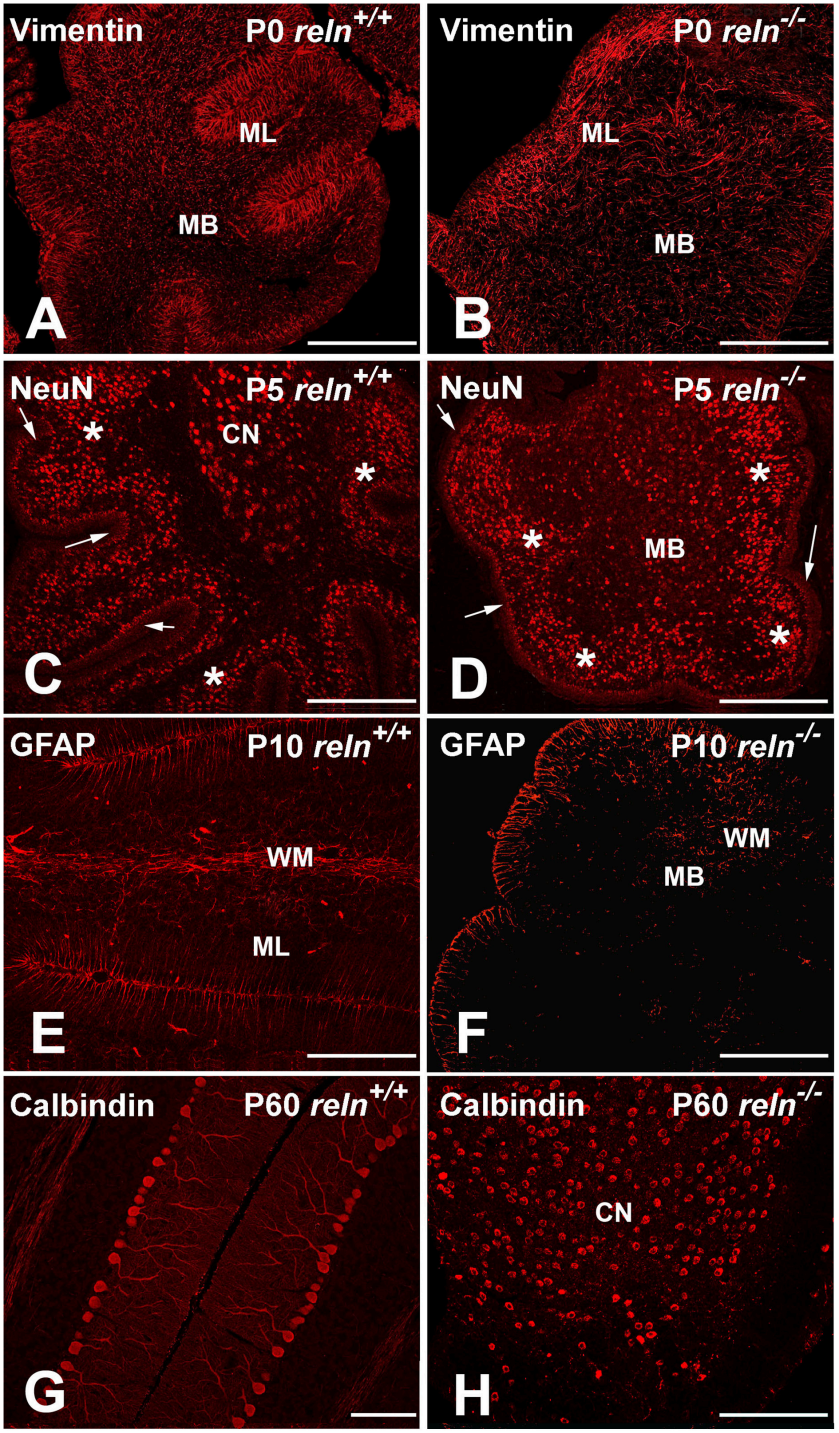
**Exemplificative images of the neurochemistry of the postnatal cerebellar cortex in normal and mutant mice**. Immunocytochemistry shows that the timing of expression of the main neuronal/glial markers in cerebellum is not affected by the *reln* mutation. **(A,B)** From P0, vimentin starts to be expressed by the Bergmann glial and the glial cells in the white matter. In the *reln*^−∕−^ ML, the Bergmann glia is poorly oriented along radial planes in comparison with controls (arrows). **(C,D)** NeuN is expressed, starting from P5, by the granule cells of the deep post-mitotic portion of the EGL (marked by arrows), the post-migratory granule cells in the IGL (asterisks) and the deep nuclei neurons (white dashed lines); **(E,F)** starting from P10, GFAP is detected in the Bergmann glia and white matter glial cells. **(G,H)** show calbindin immunoreactive Purkinje neurons in young adult mice (P60). The two images highlight the aberrant positioning of these neurons at the end of cerebellar development in *reln*^−∕−^ mice, when compared to littermate controls. In mutants, positive Purkinje neurons in the medullary body are intermingled with immunoreactive neurons of the cerebellar nuclei. BG, Bergmann glia; CN, cerebellar nucleus; EGL, external granular layer; IGL, internal granular layer; MB, medullary body; ML, molecular layer; P, postnatal age; PNs, Purkinje neurons. Scale bars: **(A–F,H)** = 100μm; **(G)** = 30 μm.

We first wanted to know if the lack of *Reelin* had direct consequences on the expression of the more general markers of neuronal and glial differentiation, as it is often assumed that development of the cerebellum in *Reeler* recapitulates—but with considerable retard—the sequences of events occurring in normal mouse. Therefore, we have analyzed the time of expression of some neurochemical markers of neural differentiation in the postnatal *Reeler* cerebellum and compared it with the pattern of expression in *reln*^+∕+^ mice (Figure 2)^1^.

The first markers to be expressed were calbindin and vimentin that could be detected at all developmental stages from birth onward. Calbindin is a specific marker of the Purkinje neurons (Schwaller et al., 2002). Calbindin^+^ Purkinje neurons in P0 *reln*^+∕+^ mice were arranged in a multi-stratified immature layer immediately below the ML. In *reln*^−∕−^ mice, calbindin^+^ Purkinje neurons were positioned instead deeply in cerebellar cortex and medullary body. Vimentin was expressed by glia (Schnitzer et al., 1981): it labels the Bergmann glia of the ML, and the glial cells of the white matter. In *reln*^−∕−^ mice, vimentin-immunoreactive (IR) Bergmann glia displayed an oblique orientation rather than being perpendicular to the pial surface of the cerebellar laminae, and were irregularly scattered in the ML.

Three other markers, NeuN, SMI32, and 67 kDa glutamic acid decarboxylase (GAD 67), tagged different populations of neurons, started to be expressed from P5, and could be subsequently detected at all other ages. Within the cerebellum, NeuN is a specific marker of post-mitotic granule cells, and its expression in these neurons positively correlates with neuritogenesis (Weyer and Schilling, 2003). SMI32, is a non-phosphorylated neurofilament protein belonging to a group of cytoskeletal proteins (Lee et al., 1988). Antibodies to SMI32 label the Purkinje neurons and the deep nuclei neurons (Milosevic and Zecevic, 1998; Leto et al., 2006). GAD 67 is a general marker of the GABAergic neurons (Greif et al., 1991). Irrespective of the genotype, NeuN positive granule cells were located in the inner part of the EGL, which harbors the pre-migratory post-mitotic subpopulation of these neurons. They were also well visible in the IGL, which becomes progressively populated by the post-migratory granule cells at the end of their migration from EGL, and in a subpopulation of neurons of the deep nuclei, which were not GAD 67-IR (see below) and, hence, use glutamate as their main neurotransmitter (Leto et al., 2006). SMI32-immmunoreactive deep nuclei neurons were embedded in the medullary body, which consisted of *bona fide* white matter in *reln*^+∕+^ mice and, in *reln*^−∕−^ mutants, of a central mass made of glial cells, myelinated fibers, granule cells, and Purkinje neurons that failed to properly migrate to the cortex. SMI32-IR deep nuclei neurons partly co-expressed NeuN (glutamatergic subgroup) or GAD 67 (GABAergic subgroup). In *reln*^−∕−^ medullary body, we could easily differentiate the SMI32-IR neurons of the deep nuclei from the Purkinje neurons using a double staining with calbindin; in *reln*^+∕+^ mice there was no need to employ SMI32+calbindin immunostaining as the two cellular populations displayed well recognizable locations in the deep gray matter and cerebellar cortex, respectively. At P5, besides to the GABAergic subgroup of the deep nuclei neurons, also the Purkinje neurons and the GABAergic interneurons in the expanding ML were GAD 67-IR.

Glial cells expressed GFAP from P10 onward. GFAP totally co-localized with vimentin in the Bergmann glia and in the white matter of the medullary body.

Remarkably, the time of expression of all markers did not show appreciable differences between *Reeler* and normal mice, and the differences that we here observed were simply related to the well-known cell mispositioning in the mutants. As marker expression is obviously linked to the differentiation status of individual cells, we could conclude that there was not a delay in the acquisition of specific cell fate markers in *reln*^−∕−^ mice. Therefore, cerebella from age-matched normal and mutant mice could be properly compared to assess the effects of the lack of Reelin.

### Hypoplasia in the *Reeler* mouse cerebellum is consequent to reduction of cortical size and cellularity, and is linked to altered temporal trends of TCD

That *Reeler* mice have a hypoplastic cerebellum almost completely devoid of folia is an established fact. What remains to be established in full are the causes of hypoplasia, as the mere impairment of neuronal migration, i.e., the primary effect of the Reelin absence, can alone hardly explain the reduction of cerebellar volume. Such a reduction can be either consequent to a diminution in the absolute numbers of neural cells and/or their density in the cerebellar cortex and nuclei (gray matter) and/or in the medullary body (white matter). In rat, the volume of the cerebellar gray matter is 3.76-fold that of the white matter (Bush and Allman, 2003) and, based on data in the cerebral cortex (Zhang and Sejnowski, 2000), ratio should reach 4.5-fold in mouse. Thus, in *Reeler* cerebellum, a reduction of TCD in the gray matter can be the primary consequence of the mutation. If such a reduction accompanies with a decrease in the area of the gray matter, then one can infer that the cerebellar hypoplasia of the *Reeler* mouse also follows the diminution in number of the cortical neurons. Another point of attention is that not only the *Reeler* mutation is responsible for hypoplasia and lack of foliation, but also it profoundly affects the cerebellar cytoarchitecture. Whether deficits are uniformly distributed or rather preferentially hit specific lobes or lobules remains to be fully ascertained. In addition, no statistical data are available to demonstrate whether the cerebellar hypoplasia specifically affects one or more layers of the forming cerebellar cortex and/or the medullary body. In the following sections, we report the results of the experiments aiming to clarifying these issues.

#### Size

Table 1 reports data on the areas of cortical layers and the medullary body in normal and *Reeler* mice in the P0–P25 time interval. From these data, *reln*^−∕−^ mice displayed a dimensionally reduced cerebellar cortex than their age-matched controls, and such a reduction was particularly prominent in the ML and IGL. When the cerebellum matures, the ML becomes populated (for the most) by the parallel fibers (i.e., the axons of the granule cells): notably, the increase in size of the ML was 16.86-fold in *reln*^+∕+^ mice, but only six-fold in the mutants. In parallel, post-migratory granule cells populate the IGL during normal development, but, from P0 to P10, the IGL increased in size of 5.1-folds in *reln* ^+∕+^ and only 2.6-fold in *reln*^−∕−^. After P10, this cortical layer only increased slightly (1.23-fold) in *reln*^+∕+^ mice, but drastically reduced its size (to 0.62-fold) in *reln*^−∕−^ mutants. Differently from the cortex, the medullary body was larger in *reln*^−∕−^ mice than in normal animals. In normal cerebellar development, the size of the medullary body mainly reflects the progressive myelination of the axons of the Purkinje neurons that leave the cortex traveling across the white matter and reach the cerebellar nuclei, as well as the development of the afferent and efferent fibers entering or exiting the cerebellum. The size of the medullary body increased in parallel with postnatal age in both genotypes (*reln*^−∕−^ 2.59, *reln*^+∕+^ 1.93-fold), but, at P25, *Reeler* mice resulted to have a larger medullary body than their normal counterparts (1.88-fold). Thus, *Reeler* mice had a smaller cerebellar cortex but a larger medullary body than their normal littermates.

**Table 1:** **Statistical analysis of the areas of cortical layers and medullary body**.

**Age**	**Genotype**	**Cerebellar cortex**			**Medullary body**
		**EGL**	**ML**	**IGL**	
P0	*reln*^+∕+^	0.07±0.007	0.07 ± 0.008	0.26 ± 0.035	0.31 ± 0.055
	*reln*^−∕−^	0.06 ± 0.014	0.03 ± 0.004	0.19 ± 0.013	0.31 ± 0.021
P5	*reln*^+∕+^	0.34 ± 0.041	0.25 ± 0.029	0.54 ± 0.043	0.30 ± 0.025
	*reln*^−∕−^	0.09 ± 0.007	0.05 ± 0.005	0.44 ± 0.026	0.42 ± 0.030
P10	*reln*^+∕+^	0.48 ± 0.019	0.80 ± 0.064	1.32 ± 0.101	0.50 ± 0.028
	*reln*^−∕−^	0.07 ± 0.006	0.07 ± 0.005	0.50 ± 0.053	0.48 ± 0.035
P15	*reln*^+∕+^	0.14 ± 0.026	1.40 ± 0.112	1.61 ± 0.099	0.62 ± 0.043
	*reln*^−∕−^	0.07 ± 0.008	0.14 ± 0.016	0.51 ± 0.032	0.64 ± 0.036
P20	*reln*^+∕+^	N/A	1.19 ± 0.102	1.13 ± 0.099	0.51 ± 0.059
	*reln*^−∕−^	N/A	0.23 ± 0.020	0.33 ± 0.025	1.09 ± 0.104
P25	*reln*^+∕+^	N/A	1.81 ± 0.169	1.63 ± 0.147	0.58 ± 0.072
	*reln*^−∕−^	N/A	0.18 ± 0.030	0.31 ± 0.050	1.09 ± 0.144

The Table reports the statistics of area measurements in the cerebellum of normal and Reeler mice (mean values in mm^2^ ± SEM). Statistically significant differences between genotypes (P < 0.05) have been indicated with gray background. EGL, external granular layer; IGL, internal granular layer; ML, molecular layer; N/A, not applicable.

#### Cellularity and its temporal variations

**Figure 3:**
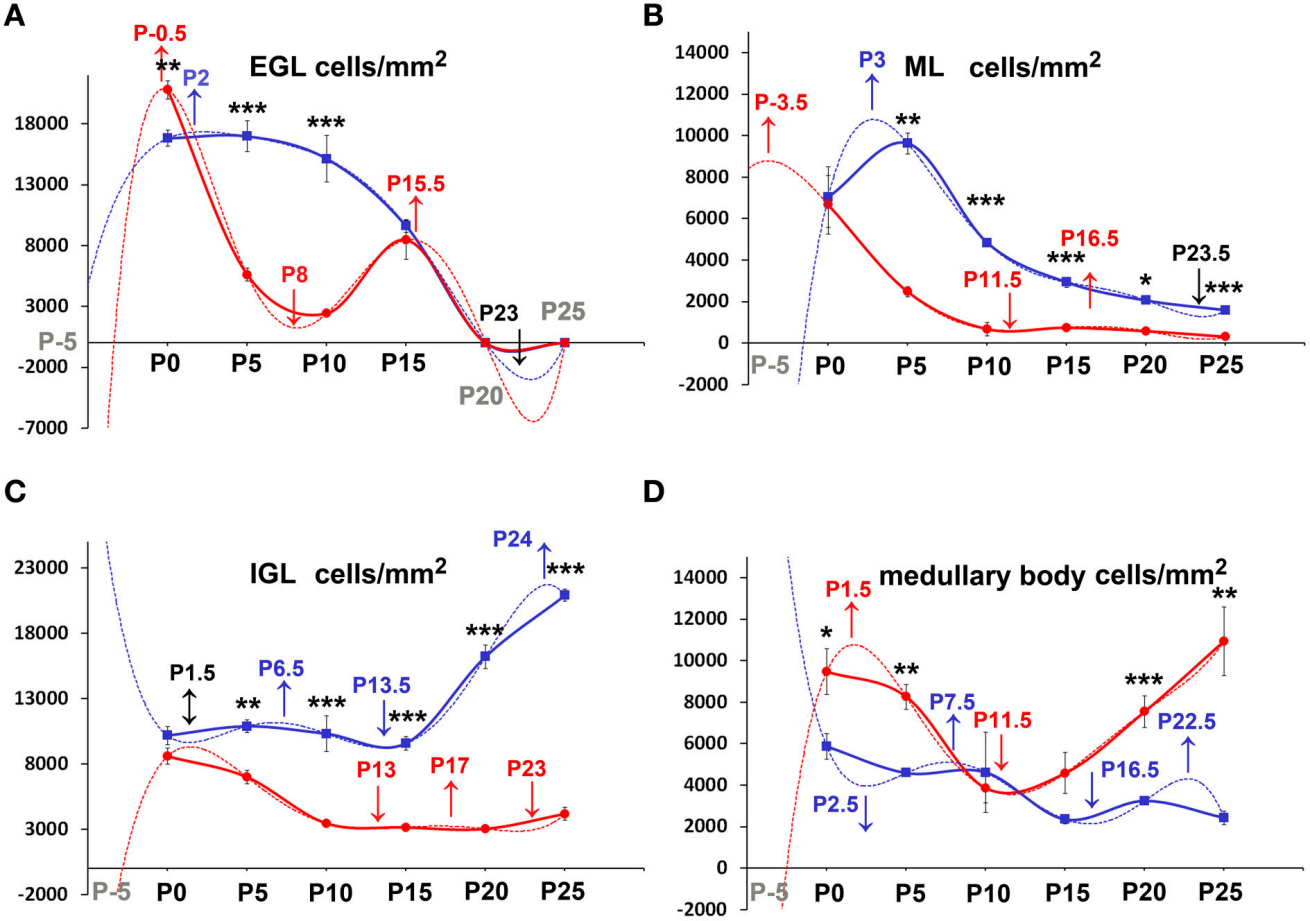
**Descriptive graphs (solid lines) and correlation curves (dashed lines) of normalized TCDs in cortical layers and the medullary body**. Graphs highlight the trends and differences *across* genotypes of normalized TCDs in the four cerebellar compartments defined in this study. Postnatal ages corresponding to the minima and maxima of correlation curves are indicated with colored letters and upwards or downwards arrows. Blue and red lines or letters/arrows refer to *reln*^+∕+^ and to *reln*^−∕−^ mice, respectively. Black letters for postnatal ages and arrows **(A–D)** refer to both genotypes. Postnatal ages in gray (x axis) indicate the time points of correlation curves outside the sampling interval (extrapolations). In *reln*^+∕+^ mice, TCD decreased in function of time after an interpolated positive peak at P2 in the EGL (**A**, blue dashed line) and at P3 in the ML (**B**, blue dashed line), whereas it increased in the IGL with an interpolated peak at P24 (**C**, red lines), and displayed an oscillatory trend in the medullary body (**D**, red lines). Although a decrease also occurred in the *reln*^−∕−^ EGL (**A**, red lines) and ML (**B**, red lines), descriptive and correlation curves were notably different when compared to those calculated for *reln*^+∕+^ mice. Extrapolated peaks at P0.5 (EGL) and P3.5 (ML) occurred earlier than in normal mice (**A**,**B**, dashed red and blue lines respectively). At P15, TCD in *Reeler* mouse EGL displayed a positive peak (**A**, solid red line), which did not have a counterpart in normal mice. The negative peaks (extrapolations) of the regression curves between P20 and P25 (in black) are predictive of the factual disappearance of the EGL in both groups of mice. In the IGL, TCD in normal *reln*^+∕+^ mice was progressively increasing (**C**, blue lines), while in *reln*^−∕−^ mice it displayed a totally opposite temporal trend, dropping dramatically over time from P5 onward (**C**, red lines). Note that, after an extrapolated minimum in *reln*^+∕+^ (blue dashed line) or maximum in *reln*^−∕−^ (red dashed line) at P1.5, a temporal switch of interpolated peaks occurs in the correlation curves of the two genotypes. Also in the medullary body **(D)** regression curves describing the correlation of TCD with time were very different in *reln*^+∕+^ (blue lines) and *reln*^−∕−^ animals (red lines). EGL, external granular layer; IGL, internal granular layer; ML, molecular layer; P, postnatal age. One-way ANOVA with multiple comparisons; error bars indicate SEM; ^***^P < 0.001; ^**^P < 0.01; ^*^P < 0.05.

**Figure 4:**
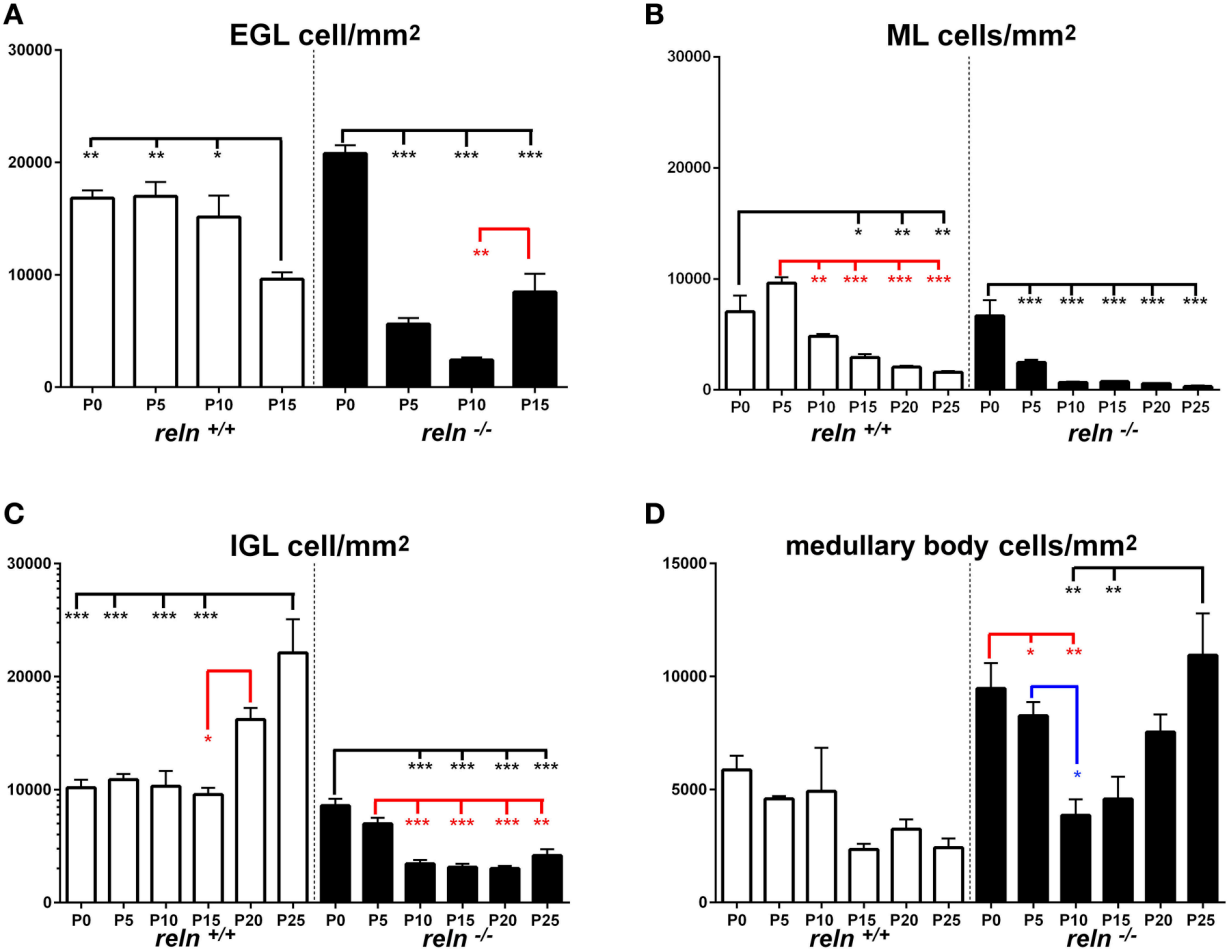
**Comparison of TCD *within* genotypes**. Cortical layers **(A–C)**, medullary body **(D)**. EGL, external granular layer; ML, molecular layer; P, postnatal age; TCD, total cell density after nuclear stain. One-way ANOVA with multiple comparisons; error bars indicate SEM; ^***^P < 0.001; ^**^P < 0.01; ^*^P < 0.05.

We then moved to consider whether and how cellularity (TCD) varied in the two groups of animals. With the Excel spreadsheet, we displayed the trend of TCD over time in line charts (Figure 3 solid lines); then, using the parametric Student T test and the non-parametric Mann Whitney test, we compared the mean values of TCD in the three cortical layers and in the medullary body between the two genotypes at corresponding postnatal ages (asterisks in Figure 3). With a One way ANOVA we have also studied TCD separately in *reln*^+∕+^ and *reln*^−∕−^ mice and compared data in relation to developmental age (Figure 4). In the EGL (Figure 3A), TCD was significantly higher in *reln*^−∕−^ mice at P0, but drastically declined at P5 and P10 to reach similar values to those of *reln*^+∕+^ mice at P15. In the ML (Figure 3B), TCD was not different across genotypes at P0, but a drastic drop occurred at P5 and P10 in *reln*^−∕−^ mice, and thus mutants displayed significantly lower values than age-matched normal mice from P5 onward. In the IGL (Figure 3C), TCD was not different among genotypes at P0. However, from P5, there was a progressive increase of cellularity in normal mice, but not in mutants. In the medullary body (Figure 3D), TCD displayed very different trends in *reln*^+∕+^ and *reln*^−∕−^ mice: in the first there was a tendency to a progressive reduction, whereas in mutants cellularity was relatively high at birth, dropped to its lowest value at P10, and increased thereafter to reach statistically significant differences with the *reln*^+∕+^ mice at P20 and P25. Figure 4 shows the results of statistical analysis comparing animals of the same genotype grouped in relation to age. This type of analysis showed that some age-related differences in TCD need to be considered with attention. For example, in the EGL of normal mice differences at P0, P5, and P10 were not statistically significant between them, as well as those at P5 and P10 in the mutants. Notably, in both genotypes TCD was lower at P15 than at birth, but in *reln*^−∕−^ mice there was a statistically significant drop at P10 (Figure 4A). As at P15 there was not a statistically significant difference in TCD between *reln*^+∕+^ and *reln*^−∕−^ mice (Figure 3A) we concluded that temporal variations in cellularity were worthy to be investigated. A similar conclusion was also drawn for the ML, IGL and medullary body after carefully inspecting the graphs in Figure 4. Therefore, we studied the temporal trend of TCD by correlation statistics.

By Pearson's correlation, we calculated the functions that best fitted our data plotting TCD against time (Table 2) and drew their graphs (Figure 3 dashed lines). Functions found to provide the best fit (*R*^2^ = 1) were 5th degree polynomial (quintic) functions. Therefore, TCD was neither constant nor varied linearly with time in the two genotypes of mice. Polynomial functions have a number of turning points at most equal to n-1, where n is the degree of the function, and each turning point is a local or global maximum (positive peak) or minimum (negative peak). Obviously, maxima and minima alternate with one another, after the curve displays an inflection point. Figure 3 clearly shows the good fit of the descriptive graphs (solid lines) with the graphs of the corresponding regression functions (dashed lines). Regression analysis allowed predicting the values of TCD continuously over time, besides the six time points of sampling. Thus, it was possible to locate temporally the turning points (i.e., the maxima and minima—indicated in Figure 3 by upwards or downwards arrows, respectively) anywhere between sampling range (interpolation) or outside this range (extrapolation). Interestingly, Pearson's correlation not only showed that relevant TCD maxima occurred earlier in the EGL and ML of *reln*^−∕−^ mice, but also that, in the P0–P5 interval, the trend of TCD was opposite to that of normal mice in the IGL and medullary body of the mutants.

**Table 2:** **Pearson's correlation equations in cortical layers and medullary body**.

**Genotype**	***f*(x)**		**Correlation equation**
*reln*^+∕+^	EGL TCD	*R*^2^ = 1	y = 106.52x^5^ − 1543.7x^4^ + 8232.5x^3^ − 21391x^2^ + 26560x + 4858
	Turning points	2	relative max: x = 1.4; relative min: x = 5.6
	EGL PrCD	*R*^2^ = 1	y = 17.342x^5^ − 210.5x^4^ + 794.46x^3^ − 1149x^2^ + 932.7x + 212
	Turning points	3	relative max: x = 2.8; relative min: x = 5.6
	EGL ApoCD	*R*^2^ = 1	y = −3.7833x^5^ + 70.728x^4^ − 494.73x^3^ + 1585.2x^2^ − 2279.7x + 1226.7
	Turning points	3	relative max: x = 2.9; x = 5.7; relative min: x = 1.6; x = 4.9
*reln*^−∕−^	EGL TCD	*R*^2^ = 1	y = 564.52x^5^ − 9342.8x^4^ + 56276x^3^ − 148897x^2^ + 160219x − 38022
	Turning points	3	relative max: x = 0.9; x = 4.1; relative min: x = 2.6; relative min x = 5.6
	EGL PrCD	*R*^2^ = 1	y = 48.712x^5^ − 783.33x^4^ + 4517.5x^3^ − 11103x^2^ + 10094x − 224.5
	Turning points	4	relative max: x = 0.7; x = 4; relative min: x = 2.6; x = 5.6
	EGL ApoCD	*R*^2^ = 1	y = 4.6211x^5^ − 77.385x^4^ + 476.4x^3^ − 1319.6x^2^ + 1595.4x − 613.48
	Turning points	4	relative max: x = 1.2; x = 4.1; relative min: x = 2.5; x = 5.6
*reln*^+∕+^	ML TCD	*R*^2^ = 1	y = 111.18x^5^ − 2172.5x^4^ + 16208x^3^ − 56631x^2^ + 88166x − 38639
	Turning points	2	relative max: x = 1.6; relative min: x = 5.7
	ML PrCD	*R*^2^ = 1	y = −0.1843x^5^ + 2.2804x^4^ − 9.606x^3^ + 32.317x^2^ − 154.4x + 322.69
	Turning points	2	relative max: x = 5.6; relative min: x = 4.3
	ML ApoCD	*R*^2^ = 1	y = −0.2909x^5^ + 6.577x^4^ − 55.16x^3^ + 210.39x^2^ − 356.96x + 224.03
	Turning points	3	relative max: x = 3.4; relative min: x = 1.8; x = 5.6
*reln*^−∕−^	ML TCD	*R*^2^ = 1	y = 31.762x^5^ − 541.92x^4^ + 3266x^3^ − 7706.6x^2^ + 3205.2x + 8417.6
	Turning points	3	relative max: x = 0.2; x = 4.3; relative min: x = 3.3; x = 5.7
	ML PrCD	*R*^2^ = 1	y = 0.1389x^4^ − 3.8335x^3^ + 36.252x^2^ − 143.28x + 202.93
	Turning points	2	relative max: x = 5.5; relative min: x = 4.3
	ML ApoCD	*R*^2^ = 1	y = 0.456x^5^ − 8.0257x^4^ + 53.04x^3^ − 162.72x^2^ + 228.34x − 110.73
	Turning points	4	relative max: x = 1.5; x = 4.1; relative min: x = 2.8; x = 5.6
*reln*^+∕+^	IGL TCD	*R*^2^ = 1	y = −193.22x^5^ + 3164.2x^4^ − 18895x^3^ + 51014x^2^ − 61543x + 36625
	Turning points	4	relative max: x = 2.3; x = 5.8; relative min: x = 1.3; x = 3.7
	IGL PrCD	*R*^2^ = 1	y = 6.7819x^5^ − 126.46x^4^ + 877.65x^3^ − 2705.6x^2^ + 3209.6x − 265.66
	Turning points	4	relative max: x = 1; x = 4.3; relative min: x = 4; x = 5.6
	IGL ApoCD	*R*^2^ = 1	y = 0.1916x^5^ − 4.9915x^4^ + 47.961x^3^ − 210.6x^2^ + 403.61x − 215.11
	Turning points	1	relative max: x = 1.9
*reln*^−∕−^	IGL TCD	*R*^2^ = 1	y = 102.78x^5^ − 1884.1x^4^ + 13023x^3^ − 41254x^2^ + 56085x − 17495
	turning points	4	relative max: x = 1.3; x = 4.4; relative min: x = 3.6; x = 5.4
	IGL PrCD	*R*^2^ = 1	y = 14.191x^5^ − 264.7x^4^ + 1870.6x^3^ − 6130.6x^2^ + 8869.9x − 3812
	Turning points	4	relative max: x = 1.5; x = 4.2; relative min: x = 3.7; x = 5.6
	IGL ApoCD	*R*^2^ = 1	y = 1.1118x^5^ − 19.114x^4^ + 123.54x^3^ − 374.31x^2^ + 526.24x − 237.61
	Turning points	4	relative max: x = 1.6; x = 3.7; relative min: x = 2.9; x = 5.6
*reln*^+∕+^	MB TCD	*R*^2^ = 1	y = −161.66x^5^ + 2802.7x^4^ − 18122x^3^ + 53867x^2^ − 73062x + 40544
	Turning points	4	relative max: x = 2.5; x = 5.5; relative min: x = 1.5; x = 4.3
	MB PrCD	*R*^2^ = 1	y = −11.207x^5^ + 193.02x^4^ − 1204x^3^ + 3257.2x^2^ − 3668.2x + 1819.1
	Turning points	4	relative max: x = 2.4; x = 5.6; relative min: x = 1; x = 4.8
	MB ApoCD	*R*^2^ = 1	y = 1.4747x^5^ − 27.548x^4^ + 193.73x^3^ − 630.95x^2^ + 924.49x − 431.46
		4	relative max: x = 1.5; x = 4.4; relative min: x = 3.2; x = 5.8
*reln*^−∕−^	MB TCD	*R*^2^ = 1	y = 102.34x^5^ − 2002.2x^4^ + 14758x^3^ − 49305x^2^ + 70263x − 24345
		2	relative max: x = 1.3; relative min: x = 3.3
	MB PrCD	*R*^2^ = 1	y = 6.0922x^5^ − 102.44x^4^ + 666.11x^3^ − 2152.6x^2^ + 3392.1x − 1347.1
		2	relative max: x = 1.9; relative min: x = 5.7
	MB ApoCD	*R*^2^ = 1	y = 3.2606x^5^ − 56.109x^4^ + 357.6x^3^ − 1039.4x^2^ + 1365x − 610.27
		4	relative max: x = 1.4; x = 4.2; relative min: x = 2.5; x = 5.6

The Table reports statistically data of correlation analysis in the three layers of the cerebellar cortex and the medullary body. The quartic describing the relation of PrCD with time is highlighted with a lighter color. All other functions are quintic. ApoCD, density of apoptotic cells; EGL, external granular layer; IGL, internal granular layer; MB, medullary body; ML, molecular layer; PrCD, density of proliferating cells; TCD, total cell density.

#### Significance

Collectively, the results reported in Sections Size and Cellularity and its Temporal Variations demonstrated the existence of important genotype-related differences in the size and cellularity (TCD) of the cerebellar cortex and the medullary body during the course of postnatal development, in parallel with profound derangements of the temporal evolution of TCD in the *Reeler* cerebellum. Specifically, the hypoplastic *reln*^−∕−^ cerebellum showed a reduction in size and cellularity of the cortex, and an increase in the medullary body. The overgrowth of the medullary body in the *Reeler* mouse is obviously a consequence of the well-known migratory defects that follow the lack of Reelin. We here quantitatively showed that such an overgrowth was insufficient to compensate the cortical hypoplasia. In addition, the concurrent reduction of the size and cellularity of the cerebellar cortex led to conclude that cortical neurons were less numerous in the mutants, as they did not display discernable differences in size. We have in fact used TEM to measure the size of the granule cells, which are—by several orders of magnitude—the most numerous cells in cerebellum, without detecting notable variations between mice of different genotypes (data not shown).

In *Reeler* mice, the alterations in size and cellularity leading to the hypoplasia of the cerebellar cortex were not uniform among layers, and the deficit in cortical growth substantially depended on reduction of the size of the ML and cellularity of the IGL. The granule cell precursors and the pre-migratory granule cells are tightly packed spheroid cells with no or very little intermingled neuropil in the EGL. Previous TEM studies in our laboratory did not show obvious ultrastructural differences in the EGL of normal and mutant mice (Castagna et al., 2014). Therefore, the higher value of TCD in the *reln*^−∕−^ EGL at birth indeed reflected the existence of a larger population of granule neurons in the mutants. The lower TCD and number of granule cells at P5–P10 (when the EGL area was also significantly reduced in *reln*^−∕−^ mice) indicated that the population of granule cell precursors was more slowly expanding in *Reeler* mice than in age-matched control animals.

Differently from the EGL, the ML is for the most occupied by an expanding neuropil that consists of the parallel fibers (the axons of the granule cells), the climbing fibers, the mossy fibers and the dendrites of the Purkinje neurons. At the same time, as the neuropil increases its size, inhibitory interneurons, i.e., the basket and the stellate cells, migrate in an outward direction from the medullary body to populate the forming ML (Miale and Sidman, 1961). Not surprisingly, at birth, when the neuropil is still poorly developed, TCD was not different across genotypes. However, as the area of the ML was smaller in *reln*^−∕−^ mice, the number of cells should be lower in the mutants, this reflecting the well documented defects in the migration of granule cells across this layer. It was also not surprising that TCD decreased with time in both genotypes but remained significantly lower in *Reeler* mice, as this reflected the drastic reduction of synaptic contacts between the Purkinje neurons and the parallel or the climbing fibers in the mutants (Mariani et al., 1977; Castagna et al., 2014). Another possible explanation of hypo cellularity in the *Reeler* ML can be advanced from the results of a study on Reelin signaling in the cerebral cortex, where interneuron laminar positions depended, at least in part, by interactions with projection neurons born on the same day in neurogenesis (Hevner et al., 2004). If this holds for cerebellum, the low cellularity of ML in *reln*^−∕−^ mice could also be linked to impairment of the migration of inhibitory interneurons as a consequence of the ectopy of the Purkinje neurons.

Post-migratory granule cells progressively populate the IGL, which will eventually become the only granule cell layer of the mature cerebellum. In *reln*^−∕−^ mice, this layer also contains some of the ectopic Purkinje neurons that failed to migrate outwardly to form the Purkinje cell layer. The area of the IGL increased with time in both genotypes, although much less in the mutants. TCD, instead, increased in *reln*^+∕+^ mice and decreased in *reln*^−∕−^ mice. Therefore, the statistically significant differences in TCD across the two genotypes also demonstrated a numerical reduction of the granule cells population in this cortical layer. These observations are fully consistent with the well-documented action of Reelin on granule cells' migration, and the previously reported reduction in their density when the *Reeler* cerebellum was analyzed *in toto* (Mariani et al., 1977; Mikoshiba et al., 1980; Heckroth et al., 1989; Yuasa et al., 1993; D'Arcangelo et al., 1995; Katsuyama and Terashima, 2009).

Finally, we observed variations in the medullary body area and TCD all along the P0–P25 interval. In *reln*^−∕−^ mice, temporal variations in TCD displayed an oscillatory trend without any discernible tendency. Therefore, our observations did not permit to assess whether there were more cells and/or they were more tightly packed in the deep central mass. Both possibilities are fully consistent with the impairment of cell migration in *Reeler* mice. No clear data are available in the literature as to the eventuality that, in the mutants, there is a volumetric reduction of the white matter. However, some forms of lissencephaly with cerebellar hypoplasia in humans have been linked to a *Reln*^−∕−^ mutated genotype, and, in these pathologies, several alterations of the white matter tracts have been reported (Ross et al., 2001; Miyata et al., 2003).

### *Reeler* mice display quantitative differences in the extent of cell proliferation and apoptosis, as well as a derangements in their temporal trends during postnatal cerebellar maturation

**Figure 5:**
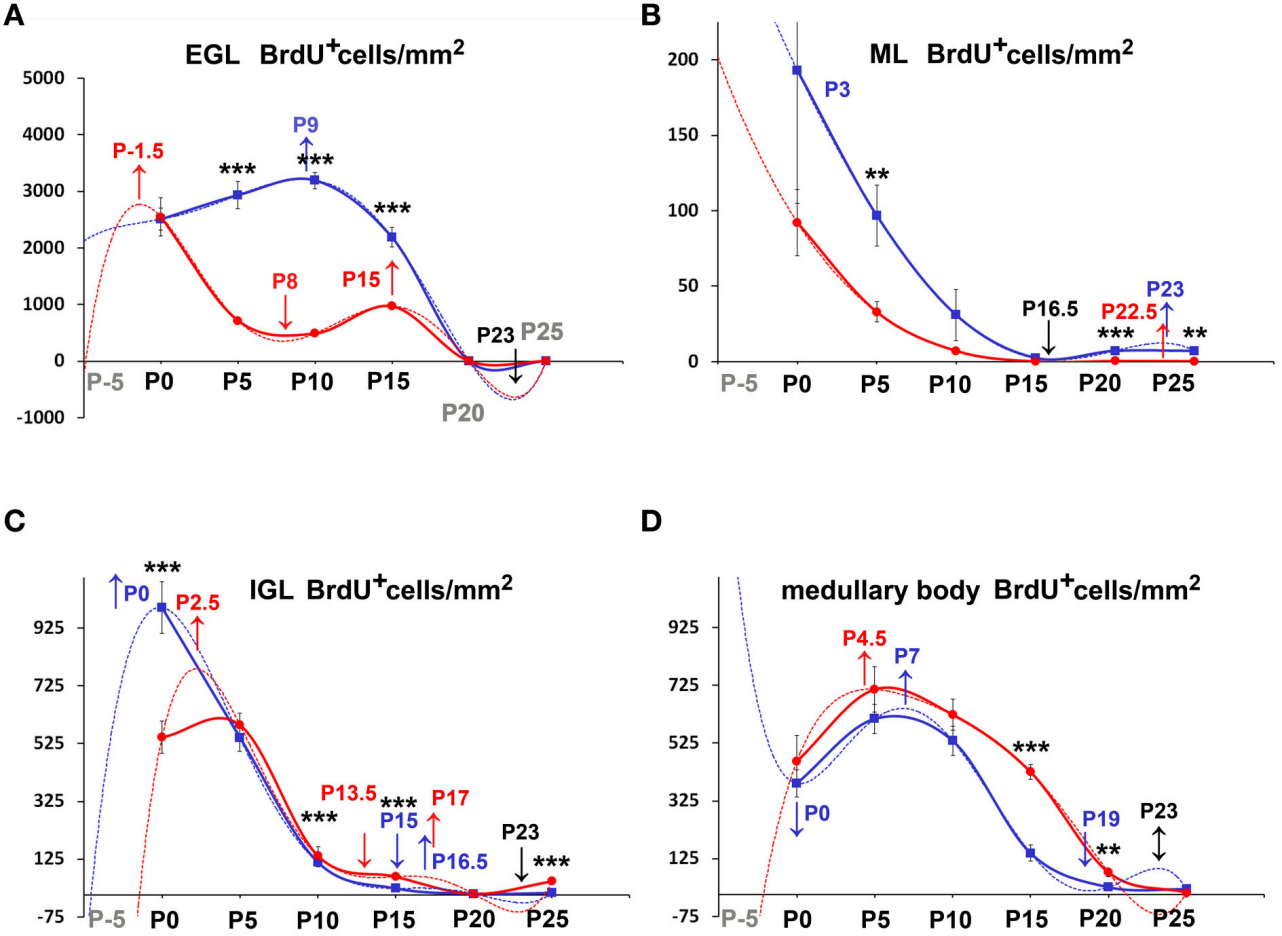
**Descriptive graphs (solid lines) and correlation curves (dashed lines) of normalized PrCDs**. Graphs highlight the trends and differences *across* genotypes of normalized PrCD (BrdU^+^ cells/mm^2^) in the four cerebellar compartments defined in this study (EGL, ML, IGL, and medullary body). Postnatal ages corresponding to the minima and maxima of correlation curves are indicated with colored letters and upwards or downwards arrows. Blue and red lines or letters/arrows refer to *reln*^+∕+^ and to *reln*^−∕−^ mice, respectively. Black letters for postnatal ages and arrows indicate reference to both genotypes. Postnatal ages in gray (x axis) indicate the time points of correlation curves outside the sampling interval (extrapolations). In the EGL, there were no significant differences across the two genotypes at birth, but, in the P5–P15 interval, PrCD was lower in the mutants (**A**, red lines). Note the temporal switch of the positive/negative peaks in the regression curves of proliferating cells in *reln*^+∕+^ (blue dashed lines) and *reln*^−∕−^ (red dashed lines) mice. In the ML **(B)**, *reln*^+∕+^ mice displayed higher PrCD (blue lines). Notably, *reln*^+∕+^ mice had a higher percentage of proliferating cells at P25 (*reln*^+∕+^ 7.16±0.01, *reln*^−∕−^ 0.25 ± 0.06, *P* < 0.01). Trends and related differences were more complex in the IGL **(C)**. PrCD was higher in *reln*^+∕+^ mice at birth, but *reln*^−∕−^ mice displayed higher values at P10, P15, and P25. Notably, in both genotypes there was a drop at P10. The percentages of BrdU^+^ cells were higher in *reln*^+∕+^ mice at P0 (*reln*^+∕+^ 9.07±0.34, *reln*^−∕−^ 6.27±0.36, *P* < 0.01), in *reln*^−∕−^ mice at P10 (*reln*^+∕+^ 1.07 ± 0.25, *reln*^−∕−^ 7.12 ± 1.41, *P* < 0.05) and P15 (*reln*^+∕+^ 0.26 ± 0.16, *reln*^−∕−^ 2.17 ± 0.41, *P* < 0.05). In the medullary body **(D)**, there were no differences in PrCD in the P0–P15 interval, and at P25. At P15 and P20, *Reeler* mice (red lines) displayed significantly higher values than *reln*^+∕+^ mice. There were not statistically significant differences in the percentages of BrdU^+^ cell. EGL, external granular layer; IGL, internal granular layer; ML, molecular layer; P, postnatal age. One-way ANOVA with multiple comparisons; error bars indicate SEM; ^***^P < 0.001; ^**^P < 0.01; ^*^P < 0.05.

**Figure 6:**
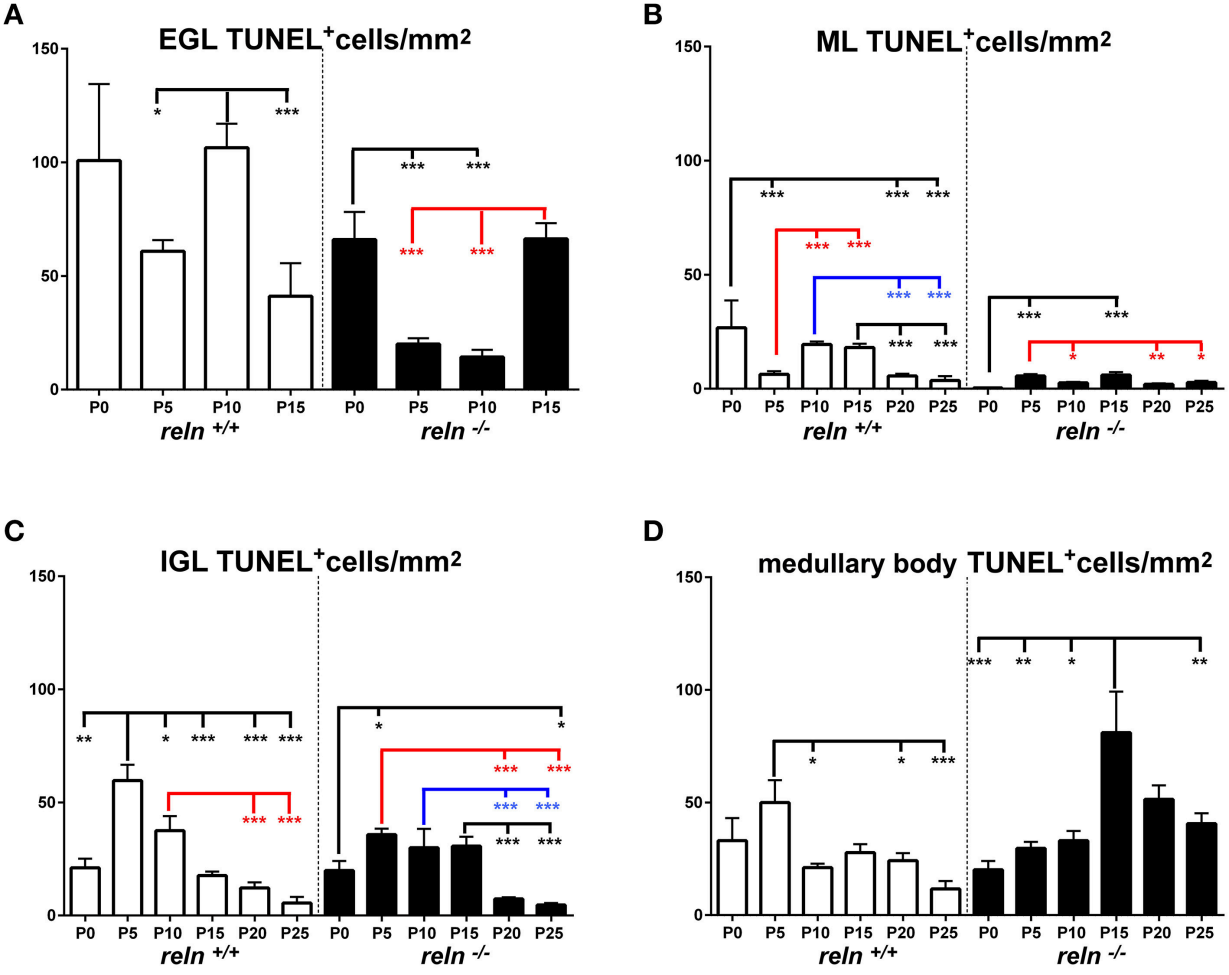
**Comparison of PrCD *within* genotypes**. Cortical layers **(A–C)**, medullary body **(D)**. EGL, external granular layer; ML, molecular layer; P, postnatal age; One-way ANOVA with multiple comparisons; error bars indicate SEM; ^***^P < 0.001; ^**^P < 0.01; ^*^P < 0.05.

**Figure 7:**
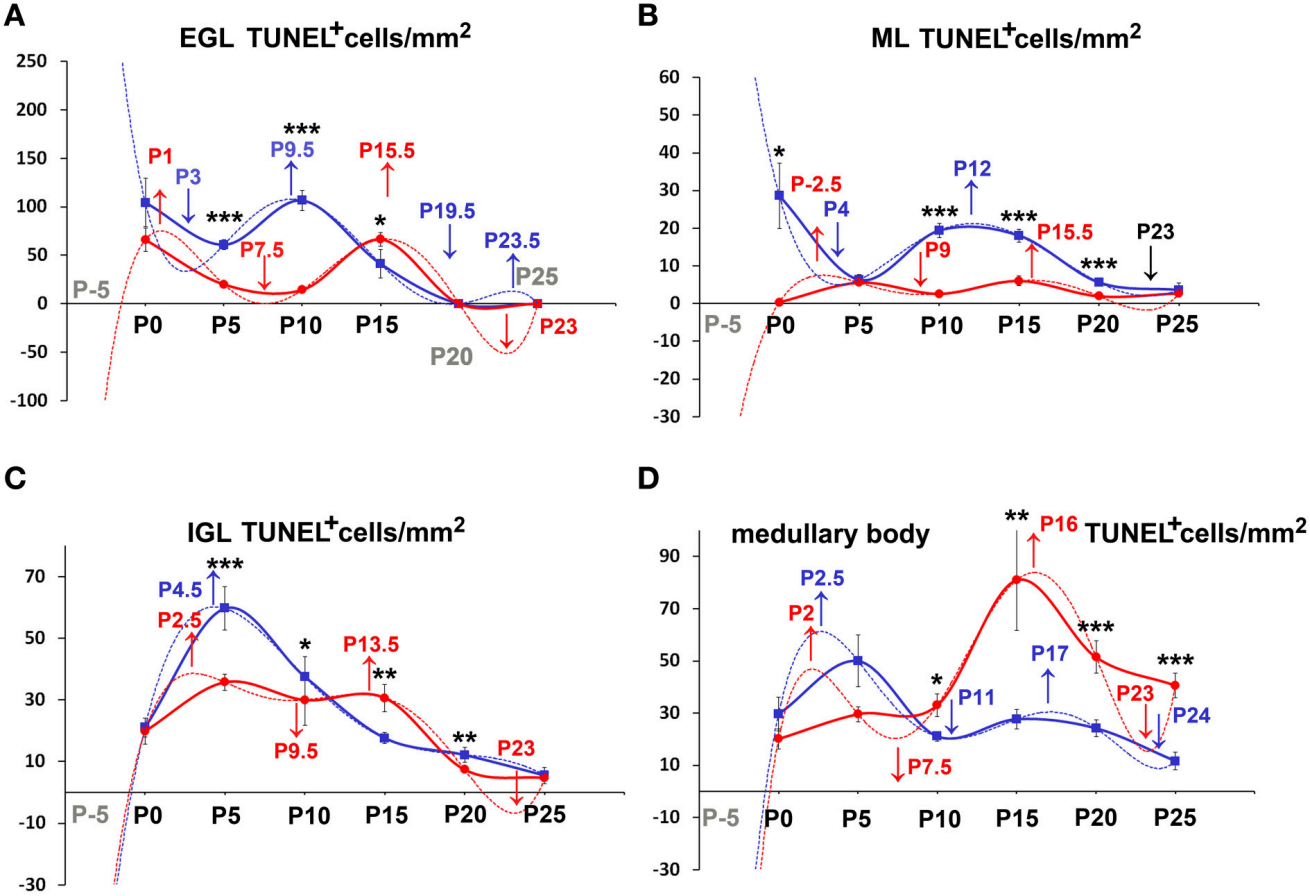
**Descriptive graphs (solid lines) and correlation curves (dashed lines) of normalized ApoCD**. Graphs highlight the trends and differences *across* genotypes of normalized ApoCD (TUNEL^+^ cells/mm^2^) in the four cerebellar compartments defined in this study (EGL, ML, IGL, and medullary body). Postnatal ages corresponding to the minima and maxima of correlation curves are indicated with colored letters and upwards or downwards arrows. Blue and red lines or letters/arrows refer to *reln*^+∕+^ and to *reln*^−∕−^ mice, respectively. Black letters for postnatal ages and arrows indicate reference to both genotypes **(B)**. Postnatal ages in gray (x axis) indicate the time points of correlation curves outside the sampling interval (extrapolations). In the EGL **(A)**, ApoCD was higher in normal mice at P5 and P10, but was then overtaken by that in *reln*^−∕−^ mice at P15, i.e., immediately before the complete disappearance of this temporary cortical layer. The percentages of apoptotic cells were not different between genotypes at any developmental age. In the ML **(B)**, *reln*^+∕+^ mice displayed higher ApoCD at P0, P10, and P20. ApoCD was not statistically different at P5 and P25, but *reln*^−∕−^ mice had a higher percentage of TUNEL^+^ cells at P5 (*reln*^+∕+^ 0.06 ± 0.02, *reln*^−∕−^ 0.23 ± 0.02, *P* < 0.001). In the IGL **(C)**, ApoCD was higher in *reln*^+∕+^ mice at P5, P10 and P20 (blue solid line), but surpassed by that calculated in mutants at P15 (red solid line). At P25, differences among genotypes were not statistically significant. The percentages of TUNEL^+^ cells were significantly higher in *reln*^−∕−^ mice only at P15 (*reln*^+∕+^ 0.18 ± 0.02, *reln*^−∕−^ 1 ± 0.15, *P* < 0.05) and P20 (*reln*^+∕+^ 0.08 ± 0.02, *reln*^−∕−^ 0.24 ± 0.03, *P* < 0.01). In the medullary body **(D)**, Apo CD was higher in *reln*^−∕−^ mice from P10 onward. There were not statistically significant differences in the percentages of TUNEL^+^ cells. In all graphs **(A–D)**, note the profound differences in the maxima/minima of correlation curves in the two genotypes. One-way ANOVA with multiple comparisons; error bars indicate SEM; ^***^P < 0.001; ^**^P < 0.01; ^*^P < 0.05.

**Figure 8:**
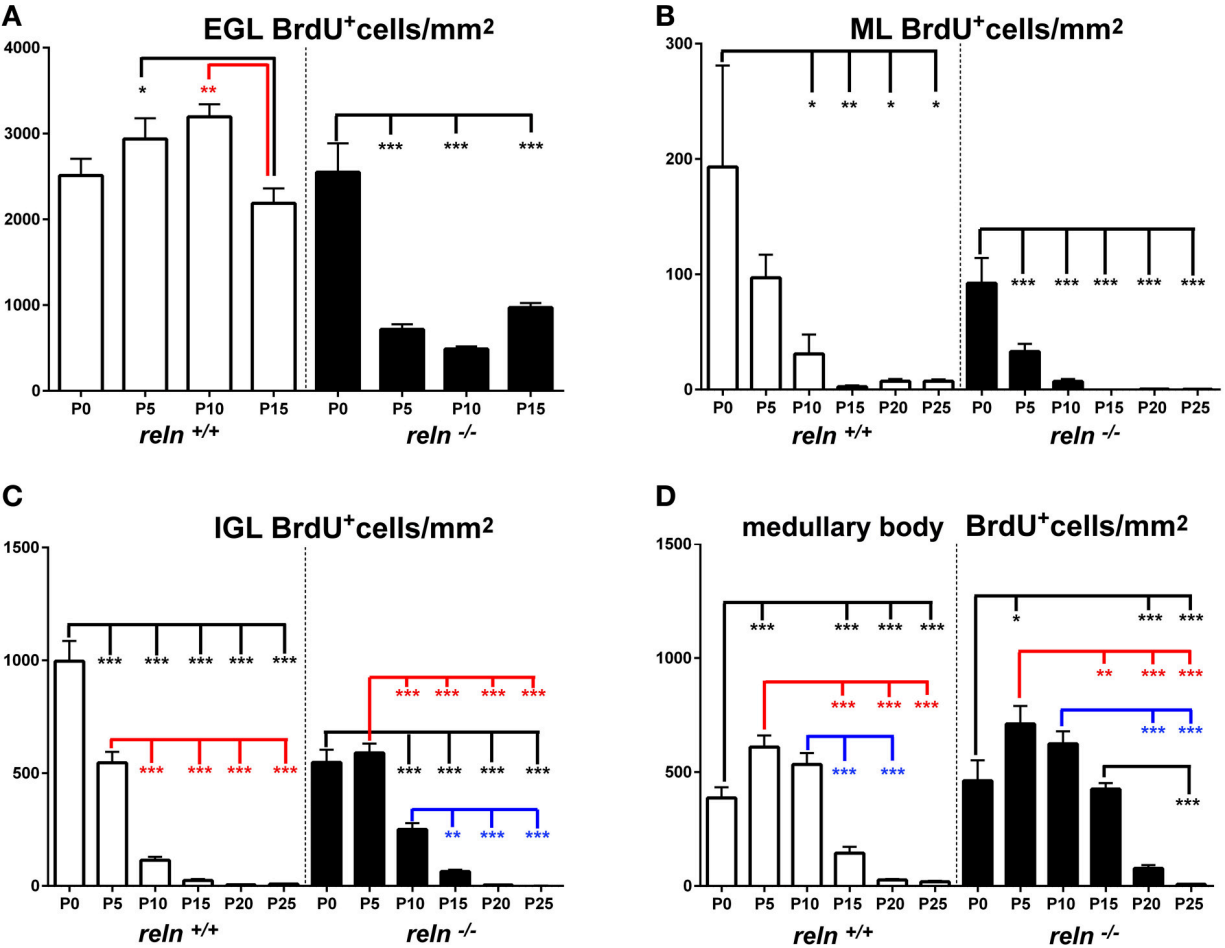
**Comparison of ApoCD *within* genotypes**. Cortical layers **(A–C)**, medullary body **(D)**. EGL, external granular layer; ML, molecular layer; P, postnatal age. One-way ANOVA with multiple comparisons; error bars indicate SEM; ^***^P < 0.001; ^**^P < 0.01; ^*^P < 0.05.

During the course of normal postnatal development, a complex interplay of cell proliferation and death is ultimately responsible for proper cerebellar maturation (Marzban et al., 2015). Therefore, we hypothesized that an imbalance between proliferation and programmed cell death could be one of the main factors to explain the cortical hypoplasia in *Reeler* mice. As apoptosis is the commonest form of programmed cell death in cerebellum (Yamaguchi and Miura, 2015), we devised a set of experiments to quantitatively investigate cell proliferation and apoptosis in our material. After a single BrdU administration, we detected high numbers of BrdU-IR nuclei in animals of both genotypes (Figures 1E,F). The number of apoptotic TUNEL^+^ nuclei was by far lower (Figures 1G,H). We have calculated the densities of the cells displaying BrdU^+^ or TUNEL^+^ nuclei and statistically analyzed data (Figures 5–**8**). To prove or disprove our hypothesis it was necessary to establish whether, above all, genotype had an influence on PrCD or ApoCD, and if additional factors, i.e., developmental age, localization in cortical layers or the medullary body, and sampling position were influential. In doing so, we first have used a 2-way repeated measures ANOVA for related, not independent groups—a type of analysis suitable for investigating changes in mean scores under three or more different conditions—to assess the influence of all these factors on PrCD or ApoCD (Section Genotype, Age, and Localization in Cortical Layers or Medullary Body—but Not Sampling Position—Affect PrC and ApoCD in the Whole Cerebellum). Then, we performed a 1-way ANOVA with multiple comparisons to establish the existence of time-related differences within each genotype and across the two genotypes at corresponding developmental ages. Finally, we used single and multiple regression analysis to model the trends of PrCD and ApoCD with time (Section Temporal Variations of PrCD and ApoCD Display a Non-Linear Relationship with Time, with Differences Across Genotypes), their reciprocal dependence, and their combined effects in determining the cellularity (TCD) of the cerebellar cortex and the medullary body (Section Regression Analysis Shows Different Non-Linear Relationships of PrCD and Apo CD Across Genotypes).

#### Genotype, age and localization in cortical layers or medullary body—but not sampling position—affect PrC and ApoCD in the whole cerebellum

According to the sampling design described in Materials and Methods, we first aimed to establish whether genotype, age, position along the latero-lateral axis of cerebellum, and localization in the cerebellar cortex or the medullary body influenced cell proliferation and/or death in the cerebellum considered in its entirety.

After a two-way ANOVA for repeated measures we found that genotype (*P* < 0.0001; *F* = 36.57; *Df* = 1), age and localization in individual cortical layers or the medullary body (age *P* < 0.0001, *F* = 25.36, *Df* = 5; cerebellar layer *P* < 0.0001, *F* = 439.89, *Df* = 3) had significant effect on PrCD values. ApoCD was also subjected to the two-way ANOVA for repeated measures and significant effects of genotype (*P* < 0.0001; *F* = 38.5, *Df* = 1), age (*P* < 0.0001, *F* = 67.54, *Df* = 5), and layer (*P* < 0.0001, *F* = 162.73, *Df* = 3) were observed. Notably, position of sampled slices along the latero-lateral axis of cerebellum had no effect on PrCD and ApoCD.

The cerebellum has a remarkably conserved architecture. With the exception of the unipolar brush cells in the vestibulocerebellum (Nunzi et al., 2001), all the other neuronal cortical types are found throughout its three major functional subdivisions, i.e., the vestibulocerebellum (cortex of the vermis+fastigial nucleus), the spinocerebellum (paravermian cortex of the hemispheres+nucleus interpositus), and the pontocerebellum (lateral hemispheric cortex+cerebellar lateral nucleus). Such a functional subdivision follows a latero-lateral axis, along which a banding pattern also occurs in relation to the expression of zebrin-II by the Purkinje neurons and the topographical distribution of the climbing and mossy fibers (Ebner et al., 2012). *Reeler* mice are ataxic, and, remarkably, in a transgenic mouse model of spinocerebellar ataxia type 8 the cerebellar banding pattern is lost, thereby contributing to the motor phenotype (Moseley et al., 2006). Therefore, absence of statistically significant difference in PrCD and ApoCD in parasagittal sections randomly sampled all along the latero-lateral axis is of biological relevance, as it demonstrated that the effect of the mutation was not related to the banding pattern of the normal mature cerebellum, but, instead, it concerned the entire organ. The spinocerebellar and vestibulocerebellar afferent projections in the *Reeler* mouse do not distribute randomly, but have specific target regions, and the position of these regions, relative to each other, is conserved in the mutants (Vig et al., 2005). The distribution of the Purkinje neurons and of the neurons of the cerebellar nuclei is not random either (Vig et al., 2005). *Reelin* binds to two high affinity extracellular receptors on the Purkinje neurons—the very low density lipoprotein receptor (Vldlr) and apolipoprotein E receptor 2 (Apoer2). In *Reeler* mice or double-null mice for Vldlr and Apoer2, Purkinje neurons' clusters failed to disperse, but animals null for either Vldlr *or* Apoer2 individually exhibited specific and parasagittally-restricted Purkinje neurons ectopias (Larouche et al., 2008). Altogether, these observations support the results of our investigation as regarding the absence of parasagittal positional differences in PrCD and ApoCD in *Reeler* mutants, as the lack of Reelin *per se* had no locally patterned influences on neuronal migration.

#### Differences in PrCD and ApoCD in relation to cerebellar architecture

Once established that sampling position was irrelevant, we proceeded to analyze the differences of PrCD (Figures 5, 6) and ApoCD (Figures 7, 8) across genotypes and within genotypes. Overall our observations demonstrated that PrCD and ApoCD were lower in the three cortical layers but higher in the medullary body of the *reln*^−∕−^ mice. A correlation analysis was then performed to study the reciprocal influences of the two parameters over time.

#### Temporal variations of PrCD and ApoCD display a non-linear relationship with time, with differences across genotypes

Figures 5, 7 show the trends of PrCD and Apo CD over time (dashed lines) and Table 2 reports their regression functions together with maxima and minima after Pearson's correlation analysis. The best fit functions (*R*^2^ = 1) were 5th degree polynomials, with the sole exception of that calculated for the ML in *Reeler*, where a 4th degree polynomial (quartic) function model was instead appropriate to describe the correlation between time and PrCD. Therefore, as it was the case for TCD (Section Cellularity and Its Temporal Variations), also PrCD and ApoCD were neither constant nor varied linearly with time, in both genotypes. It was of interest that when the regression curves describing the correlation of time with cell proliferation or apoptosis were compared in the two genotypes the following generalizations could be made: i. Proliferation/apoptotic minima and maxima were not coincident in the two genotypes; ii. Genotype-related differences in correlation curves were more evident for ApoCD; and iii. Proliferation/apoptotic maxima in *Reeler* mice generally preceded those in normal mice.

#### Regression analysis shows different non-linear relationships of PrCD and Apo CD across genotypes

There is a wide debate as to the possibility that proliferation and apoptosis are tightly interconnected during neurogenesis, as well as regarding an initial activation of mitosis as a preliminary step to apoptotic cell death (Wang et al., 2009). Therefore, we have applied regression analysis to investigate these issues. This type of analysis allows demonstrating whether a variable (predictor) is likely to be a meaningful addition to a prediction model. If this is the case (*P* < 0.05), changes in the predictor's value are related to changes in the response variable. Here we have postulated that PrCD depended on ApoCD or *vice versa*. In *reln*^+∕+^ mice, we notably found that ApoCD depended on PrCD in EGL and IGL. In *reln*^−∕−^ mice, instead, ApoCD depended on PrCD in EGL only, and PrCD depended on ApoCD in EGL and ML. Specifically, in *reln*^+∕+^ EGL ApoCD depended on (PrCD)^4^ (adjusted *R*^2^ = 1; *P* = 1,00168E-16; *Df* = 4; *F* = 5,60616E+31). In *reln*^−∕−^ EGL, ApoCD depended on (PrCD)^4^ (adjusted *R*^2^ = 1; *P* = 1.93625E-17; *Df* = 4; *F* = 1.50037E+33) and PrCD depended on (ApoCD)^4^ (adjusted *R*^2^ = 1; *P* = 8.065E-17; *Df* = 4; *F* = 8.64797E+31). In *reln*^+∕+^ IGL, ApoCD depended on (PrCD)^2^ (adjusted *R*^2^ = 0.91; *P* = 0.012248952; *Df* = 2; *F* = 26.7287703). In *reln*^−∕−^ ML, PrCD depended on (ApoCD)^3^ (adjusted *R*^2^ = 0.98; *P* = 0.011565762; *Df* = 3; *F* = 85.6282253). Collectively these observations demonstrated that predictors were different in the two genotypes, reinforcing the notion that the interdependence of PrCD and ApoCD was disturbed in the mutants.

#### Multiple regression predicts the relationship of TCD with PrCD, ApoCD and time in connection with genotype

When we studied the variations of TCD with time using Pearson's correlation (Section Cellularity and Its Temporal Variations), relationships were non-linear. Therefore, we performed polynomial regression analysis to calculate the best fit of TCD with PrCD, ApoCD or time, singularly. Table 3 reports statistically significant data. Finally, we run a multiple regression analysis modeling the relationship of TCD (response variable) simultaneously with PrCD, ApoCD and time (predictor variables). In this type of analysis, we have postulated that changes in TCD were dependent on changes in one or more of the three predictor variables: if regression was statistically significant, then it was reasonable to infer a dependency of TCD on predictor variables.

**Table 3:** **Regression analysis of TCD with PrCD, ApoCD or time**.

**Cerebellar division**	**Gen**	**Variables**		**Type of function**	**Regression statistics**			
		***y***	***x***		**Adj R^2^**	***P***	***Df***	***F***
EGL	*reln*^+∕+^	TCD	PrCD	4th deg polynomial	1	7.60734E-17	4	9.71978E+31
		TCD	ApoCD	4th deg polynomial	1	2.37729E-18	4	9.95306E+34
		TCD	Time	3rd deg polynomial	0.93	0.042	3	22.91
	*reln*^−∕−^	TCD	PrCD	Linear	0.99	2.39816E-05	1	496.860073
		TCD	ApoCD	4th deg polynomial	1	9.36107E+31	4	7.75173E-17
ML	*reln*^+∕+^	TCD	PrCD	2nd deg polynomial	0.92	0.009	2	32.05
		TCD	Time	Linear	0.72	0.02	1	13.8285904
	*reln*^−∕−^	TCD	PrCD	Linear	0.99	1.86916E-05	1	563.237209
		TCD	PrCD	3rd deg polynomial	0.98	0.008	3	120.545224
		TCD	PrCD	3rd deg polynomial	0.99	0.0036	3	273.093488
IGL	*reln*^+∕+^	TCD	Time	2nd deg polynomial	0.87	0.021	2	17.8175611
	*reln*^−∕−^	TCD	PrCD	Linear	0.85	0.005131047	1	30.877
		TCD	ApoCD	4th deg polynomial	0.99	0.022776642	4	1083.86757
		TCD	Time	2nd deg polynomial	0.90	0.012	2	26.2066774
Medullary body	*reln*^+∕+^	TCD	Time	Linear	0.75	0.015	1	16.0907388
	*reln*^−∕−^	TCD	Time	2nd deg polynomial	0.79	0.044	2	10.4717317

The Table reports statistically significant data of regression analysis in the three layers of the cerebellar cortex and the medullary body. ApoCD, density of apoptotic cells; EGL, external granular layer; IGL, internal granular layer; Gen, genotype; ML, molecular layer; PrCD, density of proliferating cells; TCD, total cell density.

In the EGL, we were unable to find a suitable model for *reln*^+∕+^ mice, although linear regression yielded data very close to statistical significance (adjusted *R*^2^ = 0.91; *P* = 0.051; *Df* = 1; *F* = 18.46307681). In mutant mice, instead, regression with PrCD (1st degree) and ApoCD (4th degree) was statistically significant (adjusted *R*^2^ = 0.99; *P* = 0.0003; *Df* = 2; *F* = 321.8028354), and coefficient statistics showed that TCD was significantly related to PrCD (P = 0.0007). In the ML, regression with PrCD (2nd degree) and time (linear) was statistically significant in *reln*^+∕+^ mice (adjusted *R*^2^ = 0.99; *P* = 0.0002; *Df* = 2; *F* = 395.3623104) and coefficient analysis showed that TCD was significantly related to both variables (PrCD^2^, *P* = 0.0009; time, *P* = 0.0138). In *reln*^−∕−^ mice linear regression statistics gave the best fit (adjusted *R*^2^ = 0.98; *P* = 0.0091; *Df* = 3; *F* = 108.4326361), and coefficient statistics showed that TCD was significantly related to PrCD (*P* = 0.0155). In the IGL, multiple regression analysis in *reln*^+∕+^ mice did not yield a suitable model to correlate TCD simultaneously with the three predictor variables of this study. In mutants, on the other hand, both linear regression (adjusted *R*^2^ = 0.97; *P* = 0.0173; *Df* = 3; *F* = 56.7676746) and polynomial regression (adjusted *R*^2^ = 0.93; *P* = 0.043; *Df* = 3; *F* = 22.52511229) were statistically significant. However, the linear model gave the worst match and coefficient statistics was not significant, whereas the polynomial regression gave a statistically significant coefficient for PrCD (*P* = 0.0098), and a value not far from significance for ApoCD^4^ (*P* = 0.07402). In the medullary body, linear and polynomial regressions were not suitable to model the relationship of TCD with PrCD, ApoCD, and time in both genotypes. Quartic functions made the interpretation less intuitive than linear or quadratic regression functions, because the effect of changing one predictor varies depending on the value of that predictor: in the temporal range of variation of this study these functions, in fact, display up to three turning points. However, these data confirmed that proliferative and apoptotic events primarily affected the cellularity of the cerebellar cortex rather than that of the medullary body, also on a predictive basis.

#### Significance

Collectively, the results reported in Sections Genotype, Age, and Localization in Cortical Layers or Medullary Body—but Not Sampling Position—Affect PrC and ApoCD in the Whole Cerebellum and Multiple Regression Predicts the Relationship of TCD with PrCD, ApoCD and Time in Connection with Genotype converged to prove that differences in cell proliferation and apoptosis in the *Reeler* mouse were more prominent in the cerebellar cortex, differently affected cortical layers, statistically influenced TCD (cellularity), and, hence, concurred to explain cerebellar hypoplasia.

##### Cerebellar cortex

The observation of important differences in PrCD and ApoCD among the layers of the forming cortex and the medullary body in normal mice was fundamental to understand that a layer-related analysis was mandatory to appreciate in full the importance of cell proliferation and apoptosis in the genesis of the mature cerebellum in *reln*^−∕−^ animals. Broadly speaking, cell proliferation, as measured by calculating PrCD, was higher in the EGL of *reln*^+∕+^ mice (Figure 5A). Therefore, taking into account the well-known impairment of granule cell migration in the mutants, it was somewhat surprising that proliferating granule cells—blocked in the EGL by the lack of Reelin—failed to increase PrCD to values higher than those calculated in *reln*^+∕+^ mice. At P5-10, migration failure of the granule cells in the mutants should theoretically lead to values of TCD higher than those recorded in normal mice; however, this was not the case (Figure 3A). Therefore, our observations demonstrated that a severely impaired cell proliferation, rather than the granule cell migratory deficit, was responsible for the differences in TCD observed in the EGL. In the ML, PrCD was also higher in normal mice (Figure 5B), but, in this case, ApoCD was higher too (Figure 7B). In normal mice, the basket/stellate cell are generated in the medullary body during the P0–P15 interval; in the same temporal window, these neurons migrate to the ML, their numerical size being regulated by apoptosis (Yamanaka et al., 2004). Therefore, lower values in the mutants likely reflected a Reelin-dependent impairment of the migration of the GABAergic interneurons. In the IGL, PrCD, and ApoCD followed a complex pattern (Figures 5C, 7C). At the end of postnatal development, PrCD was higher in the mutants, in parallel with a lower ApoCD. The GABAergic interneurons of the forming cerebellar cortex are the proliferating cells normally populating the postnatal IGL (Miale and Sidman, 1961). At birth, when the stellate and the basket cells start to be generated, PrCD was higher in *reln*^+∕+^ mice (about two-fold than in mutants). In normal mice, around P2–P7, these neurons start migrating to the ML (Miale and Sidman, 1961). We believe that migration failure explains why mutants had higher PrCD from P5. Although differences in PrCD were statistically significant, it was reasonable to suppose that the migratory deficit of the relatively small population of the GABAergic interneurons minimally reflected onto TCD. A major event was, instated, that the population of the post-mitotic post-migratory granule cells in the IGL of *reln*^−∕−^ mice underwent late apoptosis (P15 and P25 vs. P5–P10 in *reln*^+∕+^ mice) to a degree higher than in normal animals, as demonstrated by the values of ApoCD. Notably, apoptosis of mature granule cells is consequent to a failure of making proper synaptic contacts in the forming ML, and, in normal mice, programmed cell death affects IGL-migrated granule cells when synapses between the parallel fibers and the Purkinje neurons are established (Lossi et al., 2002). There are much less contacts in the mutants (Castagna et al., 2014), this explaining the higher ApoCD recorded here at P25 in *reln*^−∕−^ mice.

##### Medullary body

In the medullary body, from the second postnatal week, *Reeler* mice had higher PrCD and ApoCD (Figures 5D, 7D). However, predictive statistics failed to model TCD as a function of PrCD and ApoCD (Section Multiple Regression Predicts the Relationship of TCD with PrCD, ApoCD, and Time in Connection with Genotype). To understand the significance of these results, one needs to consider that the medullary body of the *Reeler* mouse contains the ectopic Purkinje neurons, the neurons of the cerebellar nuclei, and different types of glial cells. Apart from the Purkinje neurons, it is unclear whether the mutation also affects the migration of the other two groups of cells. Previous observations demonstrated that neurons of the cerebellar nuclei, after having been generated in the rhombic lip, migrate rostrally in a subpial stream to the nuclear transitory zone, and that a subset of rhombic lip-derived cells express Reelin (Fink et al., 2006). In later stages of development, the EGL replaces the subpial stream, and the nuclear transitory zone organizes into distinct cerebellar nuclei. It is worth noting that, in *Reeler* mice, rhombic lip-derived cells migrated normally to the nuclear transitory zone (Fink et al., 2006). Therefore, it may well be possible that the lack of Reelin does not interfere with the migration of the neurons of cerebellar nuclei. Birth-dating studies using a single pulse of BrdU (Miale and Sidman, 1961; Altman and Bayer, 1997; Sekerkova et al., 2004) indicated that most cerebellar astrocytes are generated during late embryonic and postnatal development in the prospective white matter, to which we have referred to as the medullary body in this study. Fate-restricted precursors of the astrocyte lineage reside in the postnatal prospective white matter (Cai et al., 2011), and express several markers of the juvenile astrocytes among which vimentin (Silbereis et al., 2009) that we localized in this study with overlapping patterns in the two genotypes. Whereas the interactions of Reelin with the Bergmann glia are widely documented, we were unable to find information on the intervention, if any, of the protein on the migration of immature astrocytes from the prospective white matter. It should be mentioned that some of these cells remain in the medullary body, giving rise to the white matter fibrous astrocytes, whereas others migrate to the IGL and differentiate into the velate protoplasmic astrocytes, including the bushy cells of the mature granular layer and the Bergmann glia (Palay and Chan-Palay, 1974). Thus, the higher TCD that we recorded in the *reln*^−∕−^ medullary body (Figure 3D) could also be a consequence of the entrapment of the velate protoplasmic astrocytes into the white matter because of the lack of Reelin.

## Discussion

Several spontaneous mutations primarily affecting the mouse cerebellum have been long ago discovered. They are of high interest to the neuroscientists not simply as suitable models of human ataxias (Cendelin, 2014), but also as valuable tools to study the normal development of cerebellum. Since their initial discovery, most mutants such as *Lurcher*, Purkinje cell degeneration, nervous, *Weaver*, and *Staggerer* mice were widely investigated and resulted phenotypically characterized by high levels (up to 100%) of cell death affecting the granule cells and/or the Purkinje neurons (see Table 4 in Castagna et al., 2016). Thus, mutant mice were very useful in understanding the mutual relationship between these two types of cortical neurons as regarding their reciprocal maintenance or death during the course of cerebellar maturation.

The *Reeler* mouse is somehow an exception, because programmed cell death was seldom investigated. The relative lack of interest for programmed cell death in *reln*^−∕−^ mice is not so much surprising, as the mutation was immediately recognized to be a disorder of cellular migration during the course of CNS development. The subsequent discovery of Reelin boosted a wide number of studies aiming to elucidate the cellular and molecular mechanisms that could explain its function in the normal and pathological brain (D'Arcangelo, 2014). In subsequent years, several studies described the cerebellar atrophy/hypoplasia, the disorganization of cerebellar architecture, the reduction in the number of the Purkinje neurons and density of the granule cells (Falconer, 1951; Mariani et al., 1977; Mikoshiba et al., 1980; Heckroth et al., 1989; Yuasa et al., 1993; D'Arcangelo et al., 1995; Katsuyama and Terashima, 2009). Unexpectedly, however, the only study (to our knowledge) using the TUNEL technique reported negative results (Herrup and Busser, 1995).

Here, descriptive statistics and predictive models using regression analysis proved to be useful in understanding the relationship of cellularity with proliferation, apoptosis, and time during the course of postnatal cerebellar development, disclosing the existence of notable differences between normal mice and the *Reeler* mutants. Therefore, as we will discuss below, differences in cell proliferation and apoptosis do explain, at least in considerable part, the phenotypic alterations that led to the generation of a hypoplastic cerebellum in *reln*^−∕−^ mice.

### The cerebellar cortex of the *Reeler* mouse displays altered relationship between cell proliferation and apoptosis

Especially in the EGL and IGL, *reln*^−∕−^ mice lose the link between cell proliferation and apoptosis that characterizes the normal mice; the two events are, instead, less clearly dysregulated in the ML.

In the *reln*^+∕+^ EGL, we observed a bidirectional dependence of PrCD and ApoCD that could be modeled by a 4th degree polynomial in both directions. Existence of biological phenomena that could be modeled by nonlinear regressions in the course of central neuron development is not a novel finding. For example, a 3rd degree polynomial best described pyramidal cell differentiation in layer II of the piriform cortex (Sarma et al., 2011), and hippocampal neurogenesis was adequately modeled by non-linear equations in (among others) neuronal progenitor cells and immature neurons (Cacao and Cucinotta, 2016). In addition, mathematic modeling led to better understand the complex dynamics of apoptotic regulation during brain development (Spencer and Sorger, 2011; Lavrik, 2014). Our regression statistics confirmed that cell proliferation and apoptosis of the cerebellar granule cells were tightly interconnected in normal mice, the present data being in full accordance with previous observations demonstrating that some of the newly generated granule cells in postnatal rabbits died very soon thereafter their birth, and before starting their migration to the ML/IGL (Lossi et al., 2002). Notably, such a relationship was totally lost in *Reeler* mice. In the *reln*^+∕+^ IGL, ApoCD depended on (PrCD)^2^ after regression analysis. This observation is, again, consistent with findings obtained directly by time-window labeling of proliferating rabbit granule cells that underwent a second, delayed phase of apoptotic programmed cell death after their migration to the IGL (Lossi et al., 2002). Remarkably, also this relationship was lost in *reln*^−∕−^ mice. That in *reln*^−∕−^ ML PrCD was predictively linked to (ApoCD)^3^ is more difficult to explain considering that there is not local neurogenesis in this layer. It seems possible that some granule cells in transit to the IGL remained entrapped in the ML failing to properly migrate. However, due to the high speed of migration of these cells (Zheng et al., 1996), a sequential BrdU injection protocol would be required to fully prove or disprove such a possibility. Results of predictive statistics (Section PrCD is the Most Important Predictive Factor to Determine TCD in Cortical Layers of the *Reeler* Mouse below) were also supportive of the above hypothesis.

### PrcD is the most important predictive factor to determine TCD in cortical layers of the *Reeler* mouse

After multiple regression statistics with cell proliferation, apoptosis and time as independent variables to predict TCD (cellularity), significant differences were observed among cortical layers across genotypes. However, in the EGL we were unable to find a suitable model for *reln*^+∕+^ mice, although linear regression yielded statistic data very close to significance (adjusted *R*^2^ = 0.91; *P* = 0.051). As we have only examined six time points in this study, and only four of them applied to this temporary layer of the cerebellar cortex, it seemed reasonable to hypothesize that increasing temporal sampling would yield a statistically significant model of regression. In mutant mice, multiple regression considering PrCD and ApoCD as independent variables confirmed the interdependence of the two phenomena, and coefficient statistics showed that TCD was significantly related to PrCD (*P* = 0.0007), but not to ApoCD (*P* = 0.237651133). In the ML, after coefficient analysis, TCD was significantly correlated with PrCD and time in *reln*^+∕+^ mice, but with PrCD only in *reln*^−∕−^ mice. Migration of the granule cells along the Bergmann glia is relatively rapid, as video microscopy studies *in vitro* have shown that these neurons travel at speeds between 20 and 50 μm/hour (Zheng et al., 1996). Thus, timing in migration of the granule cells may be altered in the mutants because the ML was hypoplastic, and/or migration was, itself, impaired. Finally it was remarkable that in the *reln*^+∕+^ IGL, we did not find a suitable model to correlate TCD simultaneously with cell proliferation, apoptosis and time. We interpret this finding considering the error introduced by the impossibility to quantitate cell migration according to the experimental design of this study, as the proper migration of the granule cells is of paramount importance to correctly populate the IGL. In keeping with this interpretation, in mutants, where cell migration is highly impaired, the error introduced by the regression equation was smaller and a statistically significant coefficient was calculated for PrCD (*P* = 0.0098), whereas ApoCD^4^ was not far from significance (*P* = 0.07402). Therefore, in *reln*^+∕+^ mice, the impossibility to predict the cellularity of the IGL by the predictive analysis employed in this study gave a reduction ad absurdum of the primary influence of granule cell migration in the normal development of the granular layer of the mature cerebellum.

Collectively, these predictive data confirmed the profoundly altered relationship between cell proliferation and apoptosis demonstrated after descriptive statistics in the *Reeler* mutants.

### Reelin and cell proliferation/apoptosis

The biology of Reelin in relation to positioning, growth and maturation of neurons during brain development and to synaptic activity in the adult brain has been recently and very authoritatively reviewed (D'Arcangelo, 2014). In recapitulating the history of discovery of the importance of Reelin in neuronal migration, D'Arcangelo writes “*…the layer organization of Purkinje cells in the cerebellar cortex represented the step that was directly affected by the absence of Reelin. Thus, the failure of granule cells to proliferate, which ultimately leads to the lack of foliation and cerebellar hypoplasia in reeler mice, was recognized as a secondary defect due to the malposition of Purkinje cells, which failed to enter the cerebellar cortex after leaving the ventricular zone and remained localized in a deep cerebellar mass.”* This explains why, as already mentioned in the Introduction to this paper, limited attention has been paid to the possible effects of Reelin on proliferation, and, even less, apoptosis. This work shows the existence of a deficit in granule cell proliferation as a consequence of the lack of Reelin: specifically, the very rapid temporal switch from proliferation to death in the EGL of normal mice excludes a role of mal-positioned Purkinje neurons in the target-independent apoptosis of the granule cells (Lossi et al., 2002). It seems therefore possible that the lack of Reelin directly or indirectly interferes with the proliferation program of these neurons. In keeping with this possibility, evidence is accumulating to suggest a role of Reelin in regulating cell proliferation *in vitro* (Ohkubo et al., 2007; Massalini et al., 2009) and in hippocampus *in vivo* (Duan et al., 2007; Zhao et al., 2007; Fournier et al., 2010; Teixeira et al., 2012; Sibbe et al., 2015). Our present findings are also consistent with an intervention of Reelin and/or its downstream signaling molecule Dab1 in cell death (Zhao et al., 2007; Teixeira et al., 2012). They are also in line with very recent ultrastructural findings demonstrating higher numbers of granule cells and Purkinje neurons undergoing programmed cell death in the cerebellar vermis of postnatal *reln*^−∕−^ mice in comparison with age-matched controls (Castagna et al., 2016). Current knowledge about programmed cell death has indeed accumulated toward the recognition of various different mechanisms and forms, only part of which may be subject to detection using the TUNEL technique, as we have very recently reviewed (Lossi et al., 2015). In such a scenario, it is worth mentioning that Castagna et al. (2016) have demonstrated that apoptosis is not the only type of programmed cell death occurring during postnatal cerebellar development in normal and *Reeler* mice, as autophagic neurons and neurons undergoing non-canonical forms of cell death and dark degeneration were additionally observed in both genotypes after TEM examination. To date, few data are available as regarding the possible intervention of forms of cell death other than apoptosis in cerebellar development (Marzban et al., 2015), and reported observations are often contradictory as regarding the possibility that autophagy is protective rather than harmful to cerebellar neurons. For example, the autophagy-related *Unc51.1* murine gene signals the program of gene expression leading to the formation of the granule cell axons (Tomoda et al., 1999), and the selective ablation of the *Atg5* or *Atg7* autophagy-related genes leads to behavioral deficits associated with severe neuronal loss in the cerebellar cortex (Komatsu et al., 2006, 2007). On the other hand, autophagy may be a preliminary step to granule cell apoptosis (Canu et al., 2005), and dysfunction of endosomal sorting complex required for transport 3 (ESCRT-3) causes autophagosome accumulation and neurodegeneration of the Purkinje neurons (Lee et al., 2007). Notably, the density of apoptotic neurons, irrespective of the genotype and age, was consistently higher than that of autophagic neurons in the cerebellar vermis after TEM observations (see Table 3 in Castagna et al., 2016). These observations reinforce the notion that apoptosis is the commonest form of programmed cell death not only in normal cerebellar development, also as far as the *Reeler* mouse is concerned. We might have somewhat underestimated the extent of programmed cell death in this study—if indeed autophagy of the granule cells and/or the Purkinje neurons is injurious to these neurons—and/or a switch among alternative cell death programs occurs in postnatal cerebellar development *in vivo*. Nonetheless, our observations provide a sound basis for further investigations on the intervention of Reelin in the regulation of cell proliferation in the course of (cerebellar) neurogenesis. Under this perspective, it is of relevance that the ectopic expression of Reelin in *Reeler* mice rescued animals from cerebellar ataxia, and supported a substantial recovery in granule cell proliferation (Magdaleno et al., 2002).

## Author contributions

CC performed ICC and microscopy studies, animal genotyping, descriptive statistics, and helped to draft the manuscript; AM participated in the design and coordination of the study, performed predictive statistics, critically revised statistical analysis, and drafted the manuscript; MG performed descriptive statistics; LL conceived the study, participated in its design and coordination, participated in experiments to assess cell proliferation and apoptosis, critically reviewed the manuscript draft. All authors read and approved the final manuscript.

## Funding

This work was supported by local grants of the University of Turin (Fondi ex60%).

### Conflict of interest statement

The authors declare that the research was conducted in the absence of any commercial or financial relationships that could be construed as a potential conflict of interest.

## Footnotes

^1^Outside the cerebellum, expression of these markers can change substantially as regarding to the type of labeled cells and temporal appearance. Thus, this description should be considered strictly in relation to cerebellum.

## References

[B1] Altman J. (1972a). Postnatal development of the cerebellar cortex in the rat. I. The external germinal layer and the transitional molecular layer. J. Comp. Neurol. 145, 353–398. 10.1002/cne.901450305 4113154

[B2] Altman J. (1972b). Postnatal development of the cerebellar cortex in the rat. II. Phases in the maturation of the Purkinje cells and of the molecular layer. J. Comp. Neurol. 145, 399–464. 10.1002/cne.901450402 5044254

[B3] Altman J. (1992). Programmed cell death: the paths to suicide. Trends Neurol. Sci. 15, 278–280. 10.1016/0166-2236(92)90076-K 1384195

[B4] Altman J. Bayer S. A. (1997). Development of the Cerebellar System in Relation to Its Evolution, Structure and Functions. Boca Raton: CRC Press.

[B5] Bush E. C. Allman J. M. (2003). The scaling of white matter to gray matter in cerebellum and neocortex. Brain Behav. Evol. 61, 1–5. 10.1159/000068880 12626858

[B6] Cacao E. Cucinotta F. A. (2016). Modeling impaired hippocampal neurogenesis after radiation exposure. Radiat. Res. 185, 319–331. 10.1667/RR14289.S1 26943452

[B7] Cai N. Kurachi M. Shibasaki K. Okano-Uchida T. Ishizaki Y. (2011). CD44-Positive cells are candidates for astrocyte precursor cells in developing mouse cerebellum. Cerebellum 11, 181–193. 10.1007/s12311-011-0294-x 21732075

[B8] Canu N. Tufi R. Serafino A. L. Amadoro G. Ciotti M. T. Calissano P. (2005). Role of the autophagic-lysosomal system on low potassium-induced apoptosis in cultured cerebellar granule cells. J. Neurochem. 92, 1228–1242. 10.1111/j.1471-4159.2004.02956.x 15715672

[B9] Castagna C. Aimar P. Alasia S. Lossi L. (2014). Post-natal development of the Reeler mouse cerebellum: an ultrastructural study. Ann. Anat. 196, 224–235. 10.1016/j.aanat.2013.11.004 24411683

[B10] Castagna C. Merighi A. Lossi L. (2016). Cell death and neurodegeneration in the postnatal development of cerebellar vermis in normal and Reeler mice. Ann. Anat. 10.1016/j.aanat.2016.01.010. [Epub ahead of print]. 26931496

[B11] Cendelin J. (2014). From mice to men: lessons from mutant ataxic mice. Cereb. Ataxias 1, 4. 10.1186/2053-8871-1-4 26331028 PMC4549131

[B12] Cesa R. Strata P. (2009). Axonal competition in the synaptic wiring of the cerebellar cortex during development and in the mature cerebellum. Neuroscience 162, 624–632. 10.1016/j.neuroscience.2009.02.061 19272433

[B13] D'Arcangelo G. (2014). Reelin in the years: controlling neuronal migration and maturation in the mammalian brain. Adv. Neurosci. 2014, 19 10.1155/2014/597395

[B14] D'Arcangelo G. Miao G. G. Chen S. C. Soares H. D. Morgan J. I. Curran T. (1995). A protein related to extracellular matrix proteins deleted in the mouse mutant reeler. Nature 374, 719–723. 10.1038/374719a0 7715726

[B15] D'Arcangelo G. Miao G. G. Curran T. (1996). Detection of the reelin breakpoint in reeler mice. Brain Res. Mol Brain Res. 39, 234–236. 10.1016/0169-328X(96)00046-0 8804731

[B16] Duan X. Chang J. H. Ge S. Faulkner R. L. Kim J. Y. Kitabatake Y.. (2007). Disrupted-in-schizophrenia 1 regulates integration of newly generated neurons in the adult brain. Cell 130, 1146–1158. 10.1016/j.cell.2007.07.010 17825401 PMC2002573

[B17] Ebner T. J. Wang X. Gao W. Cramer S. W. Chen G. (2012). Parasagittal zones in the cerebellar cortex differ in excitability, information processing, and synaptic plasticity. Cerebellum 11, 418–419. 10.1007/s12311-011-0347-1 22249913 PMC3856581

[B18] Falconer D. S. (1951). Two new mutants, ‘trembler’ and ‘reeler’, with neurological actions in the house mouse (*Mus musculus L.*). J. Genet. 50, 192–201. 10.1007/BF02996215 24539699

[B19] Fink A. J. Englund C. Daza R. A. Pham D. Lau C. Nivison M.. (2006). Development of the deep cerebellar nuclei: transcription factors and cell migration from the rhombic lip. J. Neurosci. 26, 3066–3076. 10.1523/JNEUROSCI.5203-05.2006 16540585 PMC6673970

[B20] Fournier N. M. Andersen D. R. Botterill J. J. Sterner E. Y. Lussier A. L. Caruncho H. J.. (2010). The effect of amygdala kindling on hippocampal neurogenesis coincides with decreased reelin and DISC1 expression in the adult dentate gyrus. Hippocampus 20, 659–671. 10.1002/hipo.20653 19499587

[B21] Gavrieli Y. Sherman Y. Ben-Sasson S. A. (1992). Identification of programmed cell death in situ via specific labeling of nuclear DNA fragmentation. J. Cell Biol. 119, 493–501. 10.1083/jcb.119.3.493 1400587 PMC2289665

[B22] Geuna S. (2000). Appreciating the difference between design-based and model-based sampling strategies in quantitative morphology of the nervous system. J. Comp. Neurol. 427, 333–339. 10.1002/1096-9861(20001120)427:3<333::AID-CNE1>3.0.CO;2-T 11054696

[B23] Greif K. F. Erlander M. G. Tillakaratne N. J. Tobin A. J. (1991). Postnatal expression of glutamate decarboxylases in developing rat cerebellum. Neurochem. Res. 16, 235–242. 10.1007/BF00966086 1780026

[B24] Hatten M. E. (1990). Riding the glial monorail: a common mechanism for glial-guided neuronal migration in different regions of the developing mammalian brain. Trends Neurosci. 13, 179–184. 10.1016/0166-2236(90)90044-B 1693236

[B25] Heckroth J. A. Goldowitz D. Eisenman L. M. (1989). Purkinje cell reduction in the reeler mutant mouse: a quantitative immunohistochemical study. J. Comp. Neurol. 279, 546–555. 10.1002/cne.902790404 2918086

[B26] Herrup K. Busser J. C. (1995). The induction of multiple cell cycle events precedes target-related neuronal death. Development 121, 2385–2395. 7671804 10.1242/dev.121.8.2385

[B27] Hevner R. F. Daza R. A. Englund C. Kohtz J. Fink A. (2004). Postnatal shifts of interneuron position in the neocortex of normal and reeler mice: evidence for inward radial migration. Neuroscience 124, 605–618. 10.1016/j.neuroscience.2003.11.033 14980731

[B28] Hirai H. Launey T. (2000). The regulatory connection between the activity of granule cell NMDA receptors and dendritic differentiation of cerebellar Purkinje cells. J. Neurosci. 20, 5217–5224. 10884305 10.1523/JNEUROSCI.20-14-05217.2000 PMC6772348

[B29] Katsuyama Y. Terashima T. (2009). Developmental anatomy of reeler mutant mouse. Dev. Growth Differ. 51, 271–286. 10.1111/j.1440-169X.2009.01102.x 19379278

[B30] Kerr J. F. Wyllie A. H. Currie A. R. (1972). Apoptosis: a basic biological phenomenon with wide-ranging implications in tissue kinetics. Br. J. Cancer 26, 239–257. 10.1038/bjc.1972.33 4561027 PMC2008650

[B31] Komatsu M. Waguri S. Chiba T. Murata S. Iwata J. Tanida I.. (2006). Loss of autophagy in the central nervous system causes neurodegeneration in mice. Nature 441, 880–884. 10.1038/nature04723 16625205

[B32] Komatsu M. Wang Q. J. Holstein G. R. Friedrich V. L. Iwata J. Kominami E.. (2007). Essential role for autophagy protein Atg7 in the maintenance of axonal homeostasis and the prevention of axonal degeneration. Proc. Natl. Acad. Sci. U.S.A 104, 14489–14494. 10.1073/pnas.0701311104 17726112 PMC1964831

[B33] Larouche M. Beffert U. Herz J. Hawkes R. (2008). The Reelin receptors Apoer2 and Vldlr coordinate the patterning of Purkinje cell topography in the developing mouse cerebellum. PLoS ONE 3:e1653. 10.1371/journal.pone.0001653 18301736 PMC2242849

[B34] Lavrik I. N. (2014). Systems biology of death receptor networks: live and let die. Cell Death Dis. 5, e1259 10.1038/cddis.2014.160 24874731 PMC4047881

[B35] Lee J. A. Beigneux A. Ahmad S. T. Young S. G. Gao F. B. (2007). ESCRT-III dysfunction causes autophagosome accumulation and neurodegeneration. Curr. Biol. 17, 1561–1567. 10.1016/j.cub.2007.07.029 17683935

[B36] Lee V. M.-Y. Otvos L. Jr. Carden M. J. Hollosi M. Dietzschold B. Lazzarini R. A. (1988). Identification of the major multiphosphorylation site in mammalian neurofilaments. Proc. Natl. Acad. Sci. U.S.A. 85, 1998–2002. 10.1073/pnas.85.6.1998 2450354 PMC279909

[B37] Leto K. Carletti B. Williams I. M. Magrassi L. Rossi F. (2006). Different types of cerebellar GABAergic interneurons originate from a common pool of multipotent progenitor cells. J. Neurosci. 26, 11682–11694. 10.1523/JNEUROSCI.3656-06.2006 17093090 PMC6674781

[B38] Lossi L. Castagna C. Merighi A. (2015). Neuronal cell death: an overview of its different forms in central and peripheral neurons, in Neuronal Cell Death, eds Lossi L. Merighi A. (New York, NY: Springer), 1–18. 10.1007/978-1-4939-2152-2 25431053

[B39] Lossi L. Mioletti S. Merighi A. (2002). Synapse-independent and synapse-dependent apoptosis of cerebellar granule cells in postnatal rabbits occur at two subsequent but partly overlapping developmental stages. Neuroscience 112, 509–523. 10.1016/S0306-4522(02)00112-4 12074894

[B40] Magdaleno S. Keshvara L. Curran T. (2002). Rescue of ataxia and preplate splitting by ectopic expression of Reelin in reeler mice. Neuron 33, 573–586. 10.1016/S0896-6273(02)00582-2 11856531

[B41] Mariani J. Crepel F. Mikoshiba K. Changeux J. P. Sotelo C. (1977). Anatomical, physiological and biochemical studies of the cerebellum from reeler mutant mouse. Philos. Trans. Royal Soc. Lond. B 281, 1–28. 10.1098/rstb.1977.0121 22882

[B42] Marzban H. Del Bigio M. R. Alizadeh J. Ghavami S. Zachariah R. M. Rastegar M. (2015). Cellular commitment in the developing cerebellum. Front. Cell Neurosci. 8:450. 10.3389/fncel.2014.00450 25628535 PMC4290586

[B43] Massalini S. Pellegatta S. Pisati F. Finocchiaro G. Farace M. G. Ciafrè S. A. (2009). Reelin affects chain-migration and differentiation of neural precursor cells. Mol. Cell. Neurosci. 42, 341–349. 10.1016/j.mcn.2009.08.006 19698788

[B44] Miale I. L. Sidman R. L. (1961). An autoradiographic analysis of histogenesis in the mouse cerebellum. Exp. Neurol. 4, 277–296. 10.1016/0014-4886(61)90055-3 14473282

[B45] Mikoshiba K. Nagaike K. Kohsaka S. Takamatsu K. Aoki E. Tsukada Y. (1980). Developmental studies on the cerebellum from reeler mutant mouse *in vivo* and *in vitro*. Dev. Biol. 79, 64–80. 10.1016/0012-1606(80)90073-1 7409324

[B46] Milosevic A. Zecevic N. (1998). Developmental changes in human cerebellum: expression of intracellular calcium receptors, calcium-binding proteins, and phosphorylated and nonphosphorylated neurofilament protein. J. Comp. Neurol. 396, 442–460. 9651004

[B47] Miyata H. Chute D. J. Fink J. Villablanca P. Vinters H. V. (2003). Lissencephaly with agenesis of corpus callosum and rudimentary dysplastic cerebellum: a subtype of lissencephaly with cerebellar hypoplasia. Acta Neuropathol. 107, 69–81. 10.1007/s00401-003-0776-0 14566414

[B48] Moseley M. L. Zu T. Ikeda Y. Gao W. Mosemiller A. K. Daughters R. S.. (2006). Bidirectional expression of CUG and CAG expansion transcripts and intranuclear polyglutamine inclusions in spinocerebellar ataxia type 8. Nat. Genet. 38, 758–769. 10.1038/ng1827 16804541

[B49] Nunzi M. G. Birnstiel S. Bhattacharyya B. J. Slater N. T. Mugnaini E. (2001). Unipolar brush cells form a glutamatergic projection system within the mouse cerebellar cortex. J. Comp. Neurol. 434, 329–341. 10.1002/cne.1180 11331532

[B50] Ohkubo N. Vitek M. P. Morishima A. Suzuki Y. Miki T. Maeda N.. (2007). Reelin signals survival through Src-family kinases that inactivate BAD activity. J. Neurochem. 103, 820–830. 10.1111/j.1471-4159.2007.04804.x 17696989

[B51] Palay S. L. Chan-Palay V. (1974). Cerebellar Cortex. Berlin: Springer Verlag.

[B52] Pujadas L. Gruart A. Bosch C. Delgado L. Teixeira C. M. Rossi D. Soriano, E.. (2010). Reelin regulates postnatal neurogenesis and enhances spine hypertrophy and long-term potentiation. J. Neurosci. 30, 4636–4649. 10.1523/JNEUROSCI.5284-09.2010 20357114 PMC6632327

[B53] Qiao S. Kim S. H. Heck D. Goldowitz D. LeDoux M. S. Homayouni R. (2013). Dab2IP GTPase activating protein regulates dendrite development and synapse number in cerebellum. PLoS ONE 8:e53635 10.1371/journal.pone.0053635 23326475 PMC3541190

[B54] Ross M. E. Swanson K. Dobyns W. B. (2001). Lissencephaly with Cerebellar Hypoplasia (LCH): a heterogeneous group of cortical malformations. Neuropediatrics 32, 256–263. 10.1055/s-2001-19120 11748497

[B55] Sarma A. A. Richard M. B. Greer C. A. (2011). Developmental dynamics of piriform cortex. Cereb. Cortex 21, 1231–1245. 10.1093/cercor/bhq199 21041199 PMC3140179

[B56] Schnitzer J. Franke W. W. Schachner M. (1981). Immunocytochemical demonstration of vimentin in astrocytes and ependymal cells of developing and adult mouse nervous system. J. Cell. Biol. 90, 435–447. 10.1083/jcb.90.2.435 7026573 PMC2111851

[B57] Schwaller B. Meyer M. Schiffmann S. (2002). ‘New’ functions for ‘old’ proteins: the role of the calcium-binding proteins calbindin D-28k, calretinin and parvalbumin, in cerebellar physiology. Studies with knockout mice. Cerebellum 1, 241–258. 10.1080/147342202320883551 12879963

[B58] Sekerkova G. Ilijic E. Mugnaini E. (2004). Time of origin of unipolar brush cells in the rat cerebellum as observed by prenatal bromodeoxyuridine labeling. Neuroscience 127, 845–858. 10.1016/j.neuroscience.2004.05.050 15312897

[B59] Sibbe M. Kuner E. Althof D. Frotscher M. (2015). Stem- and progenitor cell proliferation in the dentate gyrus of the reeler mouse. PLoS ONE 10:e0119643. 10.1371/journal.pone.0119643 25760459 PMC4356578

[B60] Silbereis J. Cheng E. Ganat Y. M. Ment L. R. Vaccarino F. M. (2009). Precursors with glial fibrillary acidic protein promoter activity transiently generate GABA interneurons in the postnatal cerebellum. Stem Cells 27, 1152–1163. 10.1002/stem.18 19418461 PMC2903623

[B61] Spencer S. Sorger P. (2011). Measuring and modeling apoptosis in single cells. Cell 144, 926–939. 10.1016/j.cell.2011.03.002 21414484 PMC3087303

[B62] Teixeira C. M. Kron M. M. Masachs N. Zhang H. Lagace D. C. Martinez A.. (2012). Cell-autonomous inactivation of the reelin pathway impairs adult neurogenesis in the hippocampus. J. Neurosci. 32, 12051–12065. 10.1523/JNEUROSCI.1857-12.2012 22933789 PMC3475414

[B63] Tomoda T. Bhatt R. S. Kuroyanagi H. Shirasawa T. Hatten M. E. (1999). A mouse serine/threonine kinase homologous to *C. elegans* UNC51 functions in parallel fiber formation of cerebellar granule neurons. Neuron 24, 833–846. 10.1016/S0896-6273(00)81031-4 10624947

[B64] Vig J. Goldowitz D. Steindler D. A. Eisenman L. M. (2005). Compartmentation of the reeler cerebellum: segregation and overlap of spinocerebellar and secondary vestibulocerebellar fibers and their target cells. Neuroscience 130, 735–744. 10.1016/j.neuroscience.2004.09.051 15590156

[B65] Wang W. Bu B. Xie M. Zhang M. Yu Z. Tao D. (2009). Neural cell cycle dysregulation and central nervous system diseases. Prog. Neurobiol. 89, 1–17. 10.1016/j.pneurobio.2009.01.007 19619927

[B66] Weyer A. Schilling K. (2003). Developmental and cell type-specific expression of the neuronal marker NeuN in the murine cerebellum. J. Neurosci. Res. 73, 400–409. 10.1002/jnr.10655 12868073

[B67] Won S. J. Kim S. H. Xie L. Wang Y. Mao X. O. Jin K.. (2006). Reelin-deficient mice show impaired neurogenesis and increased stroke size. Exp. Neurol. 198, 250–259. 10.1016/j.expneurol.2005.12.008 16438965

[B68] Yamaguchi Y. Miura M. (2015). Programmed cell death in neurodevelopment. Dev. Cell 32, 478–490. 10.1016/j.devcel.2015.01.019 25710534

[B69] Yamanaka H. Yanagawa Y. Obata K. (2004). Development of stellate and basket cells and their apoptosis in mouse cerebellar cortex. Neurosci. Res. 50, 13–22. 10.1016/j.neures.2004.06.008 15288494

[B70] Yuasa S. Kitoh J. Oda S. Kawamura K. (1993). Obstructed migration of Purkinje cells in the developing cerebellum of the reeler mutant mouse. Anat. Embryol. (Berl) 188, 317–329. 10.1007/BF00185941 7506500

[B71] Zhang K. Sejnowski T. J. (2000). A universal scaling law between gray matter and white matter of cerebral cortex. Proc. Natl. Acad. Sci. U.S.A. 97, 5621–5626. 10.1073/pnas.090504197 10792049 PMC25878

[B72] Zhao S. Chai X. Frotscher M. (2007). Balance between Neurogenesis and Gliogenesis in the Adult Hippocampus: role for Reelin. Dev.Neurosci. 29, 84–90. 10.1159/000096213 17148951

[B73] Zheng C. Heintz N. Hatten M. E. (1996). CNS gene encoding astrotactin, which supports neuronal migration along glial fibers. Science 272, 417–419. 10.1126/science.272.5260.417 8602532

